# Increased tissue tension caused by depletion of CLDN3 in the non-neural ectoderm causes neural tube defects in chick embryos

**DOI:** 10.1242/jcs.264758

**Published:** 2026-09-25

**Authors:** Elizabeth-Ann Legere, Marie Dumont, Jordan A. Dias, Yojiro Yamanaka, Gabriel L. Galea, Aimee K. Ryan

**Affiliations:** ^1^ Department of Human Genetics, McGill University, Montreal, H3A 1Y2, Canada; ^2^ Child Health and Human Development Program, The Research Institute of the McGill University Health Centre, Montreal, H4A 3J1, Canada; ^3^ Rosalind and Morris Goodman Cancer Institute, McGill University, Montreal, H3A 1A3, Canada; ^4^ Developmental Biology & Cancer Department, UCL GOS Institute of Child Health, University College London, London, WC1E 6BT, UK; ^5^ Department of Pediatrics, McGill University, Montreal, H4A 3J1, Canada

**Keywords:** Claudin, Neural tube, Tight junction, Epithelial cells, Tissue morphogenesis, Mechanics

## Abstract

Neural tube morphogenesis provides a dynamic setting in which to study epithelial cell behaviours. Here, we examined CLDN3-dependent biomechanical properties of the non-neural ectoderm during neural tube closure in chick embryos. Previous analyses of static images of chick embryos suggested that spinal neural tube closure occurs through multiple contact points or ‘buttoning’. Using live imaging and transverse cuts through the neural folds we confirmed the multiple simultaneous ‘buttoning’ contacts along the anterior-posterior axis and showed that spinal neural fold fusion is not the result of progressive ‘zippering’ cell behaviours as is required in cranial and posterior neuropore regions. CLDN3 depletion decreased the rate of neural fold closure between points of contact and increased phosphorylated myosin light chain (pMLC) within the apical domain of non-neural ectoderm cells, suggesting increased actomyosin contraction. Laser ablation studies revealed that CLDN3-depleted embryos exhibited higher tension across the non-neural ectoderm during neural fold fusion. Decreasing tension with blebbistatin rescued the neural fold fusion defects in CLDN3-depleted embryos. Our data support the importance of CLDN3 to regulate tissue tension through effects on cytoskeletal dynamics in the non-neural ectoderm during neural fold fusion.

## Teaser

**Summary:** Depletion of the tight junction protein claudin-3 (CLDN3) increases both apical actomyosin contraction and tissue tension within the non-neural ectoderm during later phases of neural tube development in chick.

## INTRODUCTION

Neural tube morphogenesis involves the coordination of complex tissue morphogenetic events that remodel a flat epithelial tissue layer into a three-dimensional tube, with the phases of neural tube development being largely conserved between species (Lee and Nagele, 1985; Sadler, 2005). In chick embryos, neural tube development begins with thickening of the neural ectoderm at the anterior midline of the embryo at ∼20 h of incubation [Hamburger-Hamilton stage (HH) 4] to form the neural plate (Hamburger and Hamilton, 1951; Schoenwolf and Alvarez, 1989). Convergent-extension movements cause the neural plate to lengthen along its anterior-posterior axis and narrow along its medial-lateral axis (Schoenwolf and Alvarez, 1989; Wallingford et al., 2002). Next, tissue at the boundary of the neural ectoderm and non-neural ectoderm elevates to form the neural folds that converge towards the dorsal midline of the embryo where the opposing neural folds meet and undergo epithelial remodelling and fusion (Lawson et al., 2001; Smith and Schoenwolf, 1989). In chick embryos, the initial site of neural fold contact occurs at the future midbrain region at stage HH8 (∼30 h), and neural fold fusion continues until stage HH12 or HH14 (∼48-52 h) in the posterior neural tube (Lawson and England, 1998). Upon fusion, the non-neural ectoderm separates from the neural ectoderm to form a continuous epithelial layer overlying the closed neural tube.

Many epithelial cell behaviours in the neural and non-neural ectoderm are required for neural tube development. These include cell neighbour exchanges during the intercalation events of convergent extension, oriented cell divisions, shrinkage of the apical cell surface during the formation of hinge points required for neural fold elevation, and, in several species, formation of filopodia and lamellipodia membrane protrusions from the non-neural ectoderm to stabilize contact between fusing neural folds (Escuin et al., 2015; Kieserman and Wallingford, 2009; Kinoshita et al., 2008; Wallingford et al., 2002). The molecular and cellular events required for the early phases of neural tube closure are relatively well understood. However, much less is known about mechanisms underlying the final phase of neural tube closure that results in neural fold fusion, especially in chick. This final phase can be broadly defined to encompass the developmental window between when the elevated neural folds complete movement to meet at the dorsal midline and the completion of the cellular remodelling events that give rise to the continuous layer of non-neural ectoderm that overlies the closed neural tube. It can be subdivided into three steps: final movements of the elevated neural folds to meet at the dorsal midline, stabilization at points of contact, and closure (Escuin et al., 2015; van Straaten et al., 2002; Zhou et al., 2020). Closure requires remodelling of cell–cell junctions, including breakdown of the junctions at the boundary of the non-neural and neural ectoderm and the re-establishment of cell junctions to unite the two sides of non-neural ectoderm into a continuous layer and to unite the two sides of the neural ectoderm to create the closed neural tube (Fig. 1A).

Of the many unanswered questions about neural fold fusion, one area of increased focus is the involvement of the non-neural ectoderm in this process. Initially, it was hypothesized that the non-neural ectoderm creates a pushing force during neural fold convergence (Alvarez and Schoenwolf, 1992). More recent data suggest that the non-neural ectoderm moves passively in response to forces generated by the neural ectoderm (Christodoulou and Skourides, 2022; Sokol, 2016), or that it may even act as an opposing force to fusion (Christodoulou and Skourides, 2022; Nikolopoulou et al., 2019). In mice, apical membrane protrusions arising from the non-neural ectoderm are hypothesized to be involved in maintaining contact between fusing neural folds. However, similar protrusions are not seen in chick embryos (Legere et al., 2024; Rolo et al., 2016). Instead, there appear to be a limited number of filopodia-like structures that are unlikely to be sufficient to maintain contact and these numbers are further reduced in CLDN3-depleted embryos (Legere et al., 2024).

After initial neural fold contact, fusion is progressively driven by movement of the closure point, the site of active fusion. This method of fusion is called ‘zippering’ and was found to be achieved by sequential cell junction shortening in *Ciona intestinalis* (Hashimoto et al., 2015). In mice, cells at the zippering point form semi-rosette structures that are dependent on interactions between integrins and the basal extracellular matrix. These rosettes, in combination with constriction of an actomyosin cable at the junction of the non-neural ectoderm and neural ectoderm, drive zippering (Molè et al., 2020; Zhou et al., 2020). In chick embryos, there is not an actomyosin cable between the non-neural ectoderm and the neural ectoderm, suggesting that alternative mechanisms must be driving neural fold fusion (Maniou et al., 2024; Pérez-Verdugo et al., 2025 preprint).

Mouse embryos are U-shaped at the onset of neural fold fusion, while most vertebrate embryos, including chick and human embryos, are flat during this stage of embryogenesis. Thus, it is expected that there will be differences in how local tissue force is generated and transmitted during neural tube development (Peeters et al., 1998a). Chick embryos also have different patterns of closure points than mice, with initial contact between neural folds occurring at the midbrain region of the embryo. Closure then progresses anteriorly until the anterior neuropore is closed, and posteriorly to an opening between the hindbrain and spinal boundary known as the rhombocervical neuropore (RCNP). Between the RCNP and the posterior neuropore (PNP), the neural folds meet at multiple points and fusion then completes almost simultaneously between these points in a process that has been referred to as ‘button-like’ or multistep closure (Fig. 1B) (Pérez-Verdugo et al., 2025 preprint; van Straaten et al., 1996, 1993; Wang et al., 2017). Closure between the most posterior point of contact in the future spinal region and the posterior end of the PNP occurs through a zippering process that is similar to that observed in mice (Jelínek et al., 1969).

To better understand the biomechanics that drive neural tube closure in chick embryos, we are studying the claudin family of tight junction proteins. Tight junctions are the most apically located cell junction and are involved in many epithelial cell behaviours, including cell–cell adhesion, maintenance of apicobasal polarity, and regulation of paracellular solute flow (Citi et al., 2024; Farquhar and Palade, 1963; Matter and Balda, 2003). The evolutionarily conserved claudin family of tetraspanins is essential for tight junction function through intra- and intercellular interactions with other claudins (Furuse et al., 1998; Krause et al., 2008; Mukendi et al., 2016). The claudin C-terminal cytoplasmic domains bridge the tight junction to cytoplasmic proteins via scaffolding proteins, including ZO1 (also known as TJP1). They have been shown to functionally interact with the cytoskeleton, apical polarity proteins, adhesion proteins, post-translational modifying proteins, and transcription factors at the tight junction cytoplasmic plaque (Hagen, 2017; Suarez-Artiles et al., 2022; Tanaka et al., 2005; Van Itallie et al., 2017). Similarly to adherens junctions, tight junctions can interact with the cortical actin network, a hallmark of the apical domain of epithelial cells (Van Itallie et al., 2017). Regulation of cortical actin underlies almost all cell morphogenetic behaviours and links claudins to actin-dependent cell behaviours during neural tube development (Escuin et al., 2015; van Straaten et al., 2002; Zhou et al., 2020).

Claudin expression is temporally and spatially restricted during all stages of development, including neural tube morphogenesis (Collins et al., 2013). Manipulating claudin expression in the non-neural or the neural ectoderm provides a mechanism to independently examine the effect of each tissue in chick neural tube development. We discovered that depletion of claudin family members from the neural or non-neural ectoderm result in neural tube defects (NTDs) due to failure of early and late phases of neural tube closure, respectively. Specifically, we showed that embryos with NTDs resulting from CLDN8 depletion from the neural ectoderm had reduced localization of the planar cell polarity protein VANGL2 in the neural ectoderm during convergent extension and altered localization of the actin-modifying proteins CDC42, RHOA and phosphorylated myosin light chain (pMLC) (Baumholtz et al., 2017). NTDs also occur when CLDN3, which is specifically expressed throughout the entire non-neural ectoderm (Collins et al., 2013; Haworth et al., 2005; Simard et al., 2006), is depleted (Legere et al., 2024). The NTDs in CLDN3-depleted embryos are due to a failure in neural fold fusion, with most occurring in the future spinal region of the embryo (Legere et al., 2024)*.* Cells within the non-neural ectoderm of CLDN3-depleted embryos have smaller apical areas and increased number of membrane blebs, a sign of unstable cytoskeletal to membrane attachments and/or increased contractility (Sedzinski et al., 2011). In addition, within the apical domain, there is an increase in the apical polarity proteins PAR3 (also known as PARD3) and MPP5, and a decrease in bicellular-localized F-actin compared to control embryos (Legere et al., 2024). In this study, we extend our study of neural fold fusion defects in CLDN3-depleted chick embryos to further investigate the role of the non-neural ectoderm in neural fold fusion and the molecular mechanisms responsible for this phase of neural tube morphogenesis.

## RESULTS

### Neural fold fusion occurs by distinct mechanisms in cranial and spinal regions of the chick neural tube

Previous analyses of static images identified multiple closure points along the anterior-posterior axis of the future spinal region of the chick embryo that suggest that this region of the neural tube does not close by zippering (van Straaten et al., 1993). To study this in real time, we performed live imaging of *ex ovo* cultured chick embryos and assessed neural fold fusion in the cranial and spinal regions (*n*=5). As expected, the first point of contact between neural folds occurred at the future midbrain region at HH8 (∼30 h) and the opposing neural folds progressively closed in the anterior and posterior directions by zippering from this point (Fig. 1C, T0-T3; Movie 1). While zippering continued in the anterior direction until the anterior neuropore was closed, the posterior-directed zippering paused within the future hindbrain region (Fig. 1C, T3-T4). Posterior to the stalled zippering point, the neural folds at the level of the first somite pair bulged towards the dorsal midline until contact was made with the opposing neural folds to form the posterior boundary of the RCNP (Fig. 1C, T3-T4). Our live imaging data showed that the RCNP formed at approximately HH9 with its posterior boundary at the level of the first somite pair in most control embryos (*n*=3/4) and at the level of the third somite pair in one embryo. We observed that the length of time it took for the RCNP to close ranged from 30 to 55 min, with the longest time attributed to the embryo where the posterior boundary of the RCNP extended to the level of the third somite pair (data not shown). We do not know if the same variability occurs *in ovo*.

**Fig. 1.**
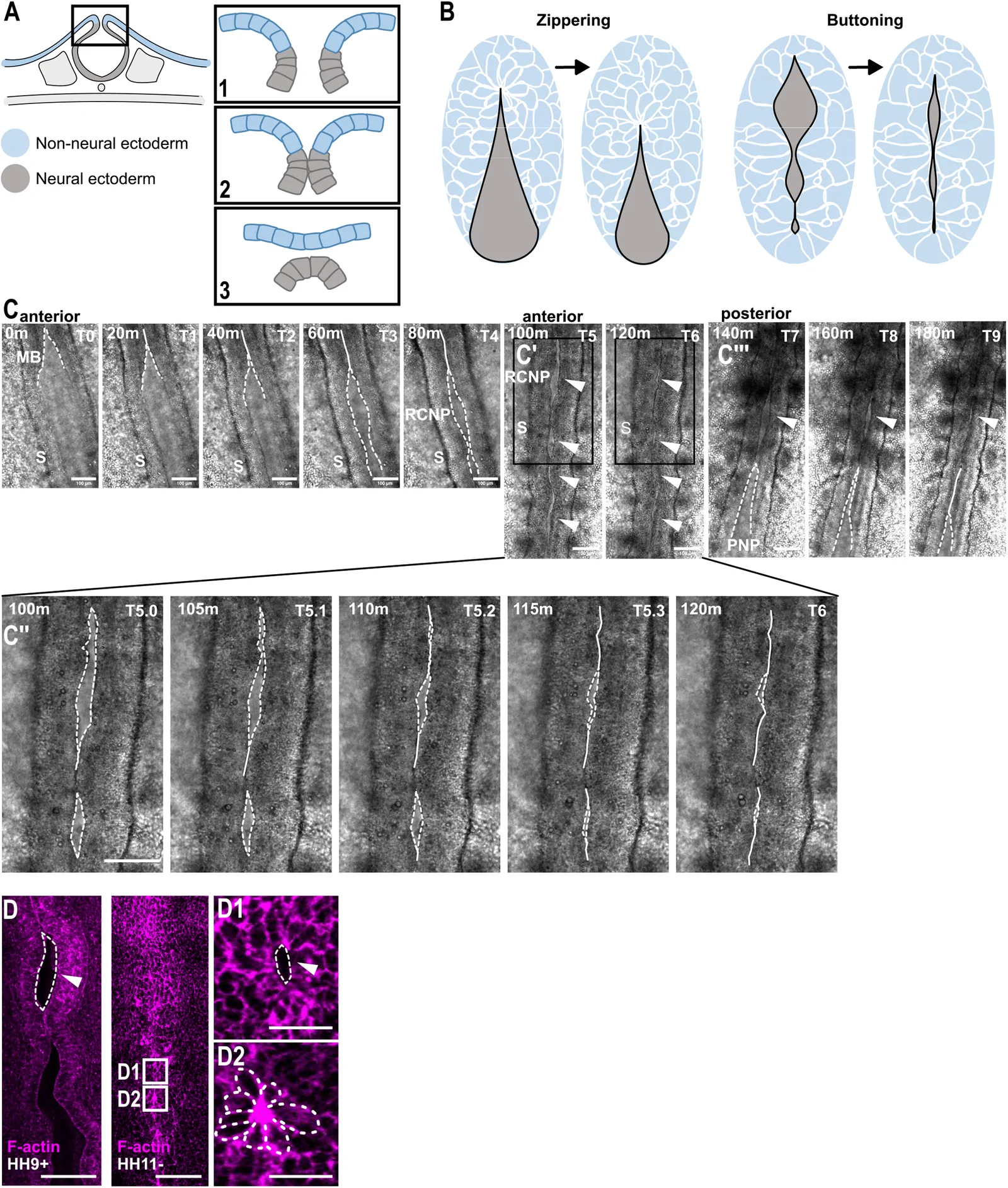
**The process of neural fold fusion is distinct between cranial and spinal regions.** (A) Schematic of a transverse view of neural fold fusion in chick embryos where the neural folds are (1) moving, (2) making contact, and (3) fused. Non-neural ectoderm: blue; neural ectoderm: grey. (B) Schematic of a dorsal view of the progression of zippering and buttoning during neural fold fusion in chick embryos. (C-C‴) Representative maximum intensity projections from live imaging of control embryos (*n*=5) at 20-min (m) intervals (T0-T9). MB, midbrain; PNP, posterior neuropore; RCNP, rhombocervical neuropore; S, first somite. White dashed lines outline the edges of unfused neural folds, and white solid lines mark the midline of fused neural folds. White arrowheads mark gaps between button points (Movie 1). In C′, imaging was paused to adjust focus (Movie 2). Black boxes mark regions shown in higher magnification stills in C″ at 5-min intervals (T5.0-T6.0). In C‴, imaging was stopped to readjust the position of the embryo to image the posterior region of the future spine. (D) Representative maximum intensity projection images of HH9 (*n*=3) and HH11 (*n*=4) embryos stained with phalloidin (F-actin; magenta). Boxed areas are shown at higher magnification in D1,D2. White dashed lines outline the edges between open neural folds and dashed lines outline cells surrounding neural fold fusion in D2. Arrowheads in D and D1 indicate gaps between button points. Scale bars: 100 μm (C-C‴,D); 50 μm (D1,D2).

Live imaging also revealed the almost simultaneous appearance of multiple additional smaller bulges along the anterior-posterior axis of the spinal neural folds to create the multiple ‘buttoning’ contact points previously observed in static images taken during chick neural fold fusion (Lawson and England, 1998; van Straaten et al., 1996, 1993). The gaps between these button contacts then appeared to close independently within the next 20-30 min through lateral motion of the neural folds, producing thin and elongated gaps (Fig. 1C′, T5-T6; 1C″; Movie 2). The most posterior contact point formed the anterior boundary of the PNP and from this point the neural tube fused with stereotypical posterior-directed zippering (Fig. 1C‴, T7-T9). We confirmed that the RCNP and multiple openings along the spinal region of the embryo were present *in ovo* (*n*=8) (Fig. S1A) and in *ex ovo* cultured chick embryos (*n*=29) (Fig. S1D), and that both the non-neural ectoderm and the neural ectoderm were unfused within the gaps (*n*=3) (Fig. S1B).

By combining the live imaging data with confocal images of ZO1 immunofluorescence to identify the lateral cell membranes, we defined three steps that occur during buttoning: initial contact between the neural folds at button points to form gaps, movement of the neural folds to make contact between the button points, and closure of the gaps. We and others observed that beginning at HH8 the elevated unfused neural folds appeared ‘wavy’ (Fig. 1C,D) (Maniou et al., 2024). Our data also suggested that the wavy shape of the neural folds is not driven by specific actomyosin constriction at the neural fold edge, as there was no enrichment of F-actin at unfused neural fold edges compared to the expected enrichment of F-actin where neural folds are in contact and actively fusing (*n*=7) (Fig. 1D). Our analysis of phalloidin-stained embryos aligned with previous observations that chick embryos do not have a supracellular actin cable between the non-neural and the neural ectoderm (Maniou et al., 2024). We observed that the gaps closed progressively from both the anterior and posterior directions until only a small area in the centre remained (Fig. 1C″,D1). Whole-mount immunofluorescence images of ZO1 expression support that the neural folds moved progressively towards each other to close the gaps (*n*=3) (Fig. S1C1), and then the cells pinched together (Fig. S1C2) to form a rosette-like shape to complete fusion and close the opening (Fig. 1D2; Fig. S1C3).

To determine whether the zippering and buttoning movements were independent of each other, we performed similar experiments to those previously used to evaluate neural fold zippering in *Ciona* (Hashimoto and Munro, 2019). When *Ciona* embryos are cut in half during neural fold zippering, the half that contains the point of contact (zippering point) completes neural fold fusion as normal, while the half that does not contain the zippering point does not (Hashimoto et al., 2015). We adapted this method for the chick embryo to test whether buttoning depends on zippering occurring in the cranial region. Briefly, chick embryos were cultured until the appropriate stage, the vitelline membrane was removed, and then a hair-knife was used to cut the embryos at one of three locations: posterior to the first closure point in the midbrain, at the level of the fifth somite pair, or within the PNP. Cut embryos were cultured dorsal side-up sandwiched between two filter papers for up to 24 h and assessed for neural tube closure defects (Fig. 2). The spinal regions of all embryos cut posterior to the first closure point in the midbrain at HH8 were able to fuse and complete neural tube closure (*n*=7) (Fig. 2A,B). Similarly, when the forming neural tube was cut at the level of the fifth somite pair in HH9 embryos, all embryos were able to complete spinal neural fold fusion (*n*=9) (Fig. 2A,B). These data suggest that, unlike zippering in *Ciona* which depends on the behaviour of cells at the zippering point, buttoning of the chick neural folds is independent of the presence of the zippering point or continuous zippering.

**Fig. 2.**
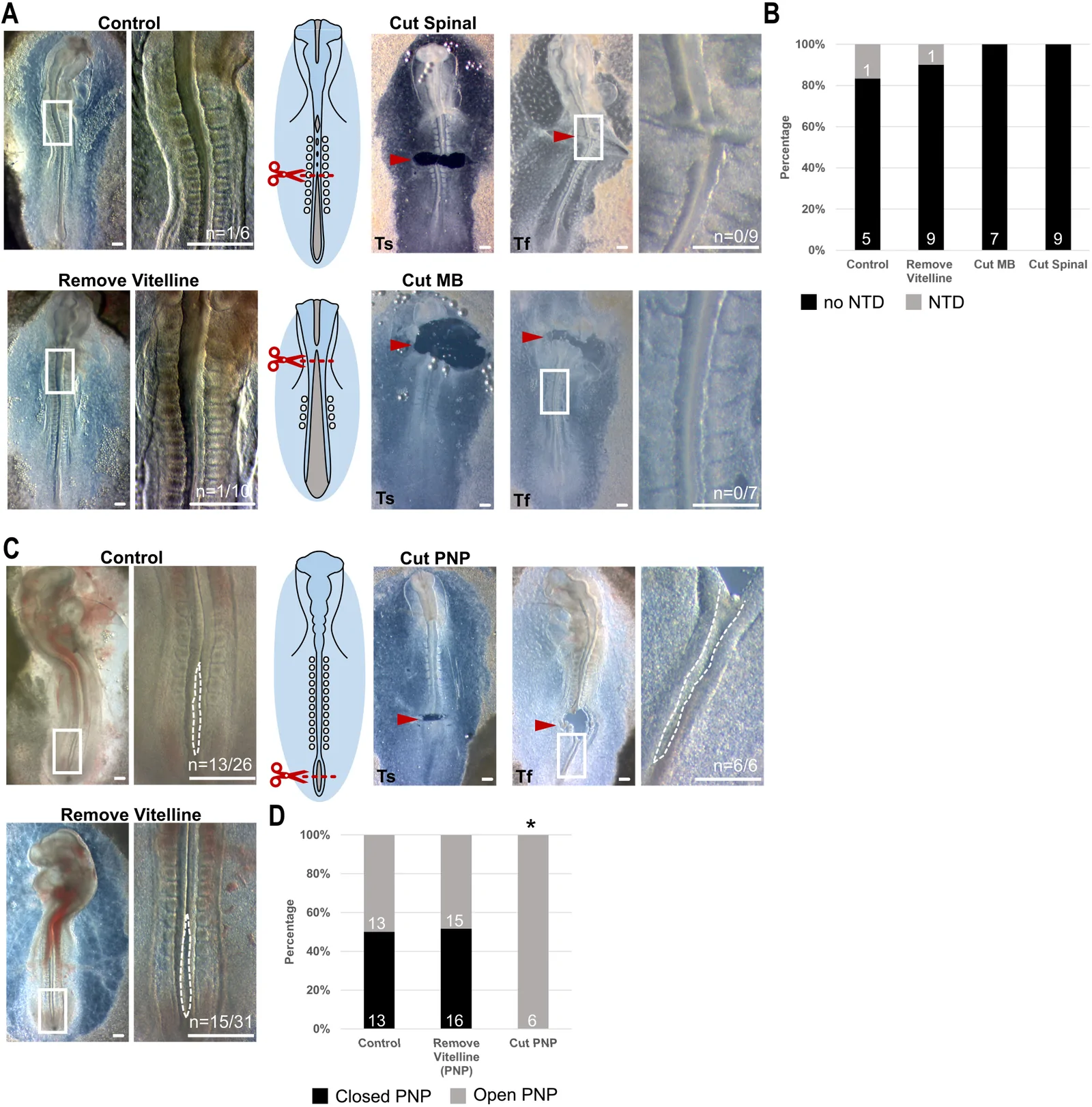
**Buttoning is independent of zippering.** (A) Representative images of control embryos (non-manipulated) (*n*=6), ‘Remove Vitelline’ embryos (*n*=10) (vitelline membrane removed over spinal region), and embryos cut at the midbrain (MB) (*n*=7) and at the level of the fifth somite pair in the mid-spine region (‘Spinal’) (*n*=9) without NTDs. The number of embryos with NTDs/total is in the lower right corner. Schematics of the cutting experiment are shown on the left of the representative images of cut embryos. Images were taken immediately after cutting at the start of culturing (Ts) and when the culturing period was finished (>15 h; Tf). White boxes outline regions shown in higher magnification images. Red arrowheads mark cut sites. (B) Quantification of the percentage of NTDs in control embryos, uncut embryos with vitelline membrane removed over spinal region (Remove Vitelline) and cut embryos (Cut MB and Cut Spinal). (C) Representative images of control (*n*=26), Remove Vitelline (*n*=31) (removed over PNP) and embryos cut through the PNP (*n*=6) with NTDs. White dashed lines outline open neural folds in the PNP. (D) Quantification of the percentage of NTDs in the PNP (open and closed PNP) in control (uncut embryos), uncut embryos with vitelline membrane removed over the PNP (Remove Vitelline) and cut embryos (Cut PNP). Statistical significance was evaluated with Fisher's Exact Test. *P<0.05. Scale bars: 200 μm.

To confirm that zippering would not progress without the presence of a zippering point, we cut older (HH10-HH11) embryos within the middle region of the PNP and allowed them to develop until HH14 and then assessed whether fusion of the PNP occurred, expecting some controls to have PNP defects due to the sandwiched filter paper culture method (Kunz et al., 2023). All cut embryos contained neural fold fusion defects in the PNP (*n*=6/6) compared to 52% of uncut stage-matched control embryos that had their vitelline membrane removed over the PNP region (*n*=15/31, *P*=0.02) or embryos that were cultured similarly without removal of the vitelline membrane (*n*=13/24, *P*=0.05) (Fig. 2C,D).

### CLDN3 depletion decreased the rate of neural fold movement between button points during fusion

Given that NTDs in CLDN3-depleted embryos occur primarily in the future spinal region (Legere et al., 2024), we examined whether there were visible differences during spinal neural fold fusion between control and CLDN3-depleted embryos. We previously reported that CLDN3-depleted embryos form dorsolateral hinge points and do not have a delay in closure of the PNP (Legere et al., 2024). To be able to image embryos midway through neural fold fusion (HH8-HH10), embryos were cultured *ex ovo* on agar-albumen plates (Chapman et al., 2001) containing either the GST-C-CPE (C-terminal domain of *Clostridium perfringens* enterotoxin) variant GST-C-CPE^LDR^ (LDR) (400 μg/ml) that specifically binds and removes CLDN3 from tight junctions or in molarity-matched glutathione S-transferase (GST; 266 μg/ml) as control (Legere et al., 2024; Veshnyakova et al., 2012). We refer to LDR-treated embryos as CLDN3-depleted and the GST-treated embryos as control embryos throughout the text, and use LDR and GST labels, respectively, in figures. There were no observed changes in the initial formation of button contact points and no evidence of button ‘mismatches’ as determined by measuring the percentage average difference between the number of cells bordering the left and right sides of the open gap between button points in control (*n*=4) and CLDN3-depleted (*n*=4) embryos (GST: 14±11%; LDR: 16±10%; mean±s.d.; *P*=0.86) (Fig. S2A,B).

The RCNP is the gap formed between the end of zippering in the cranial region and the most anterior button point in the spinal region. It appears to close similarly to the gaps between button points in the spinal region and its larger size makes it more amenable for analysis. Therefore, we used the RCNP to measure whether there was a change in convergence of the neural folds to close the RCNP gap. We first measured the area and perimeter of RCNPs of stage-matched HH9 control (*n*=4) and CLDN3-depleted (*n*=5) embryos and found that in CLDN3-depleted embryos the RCNP areas (GST: 448.0±354.5 μm^2^; LDR: 2802.6±979.1 μm^2^; mean±s.d.; *P*=0.003) and perimeters (GST: 117.2±22.0 μm; LDR: 277.0±46.5 μm; *P*=0.0005) were larger (Fig. 3A,B). There was no change in the ratio of anterior-posterior to medial-lateral RCNP lengths between control and CLDN3-depleted embryos (GST: 2.9±1.2 μm; LDR: 2.9±0.4 μm; mean±s.d.; *P*=0.96) or RCNP circularity (GST: 0.36±0.2; LDR: 0.44±0.1; *P*=0.48) (Fig. S2C,D).

**Fig. 3.**
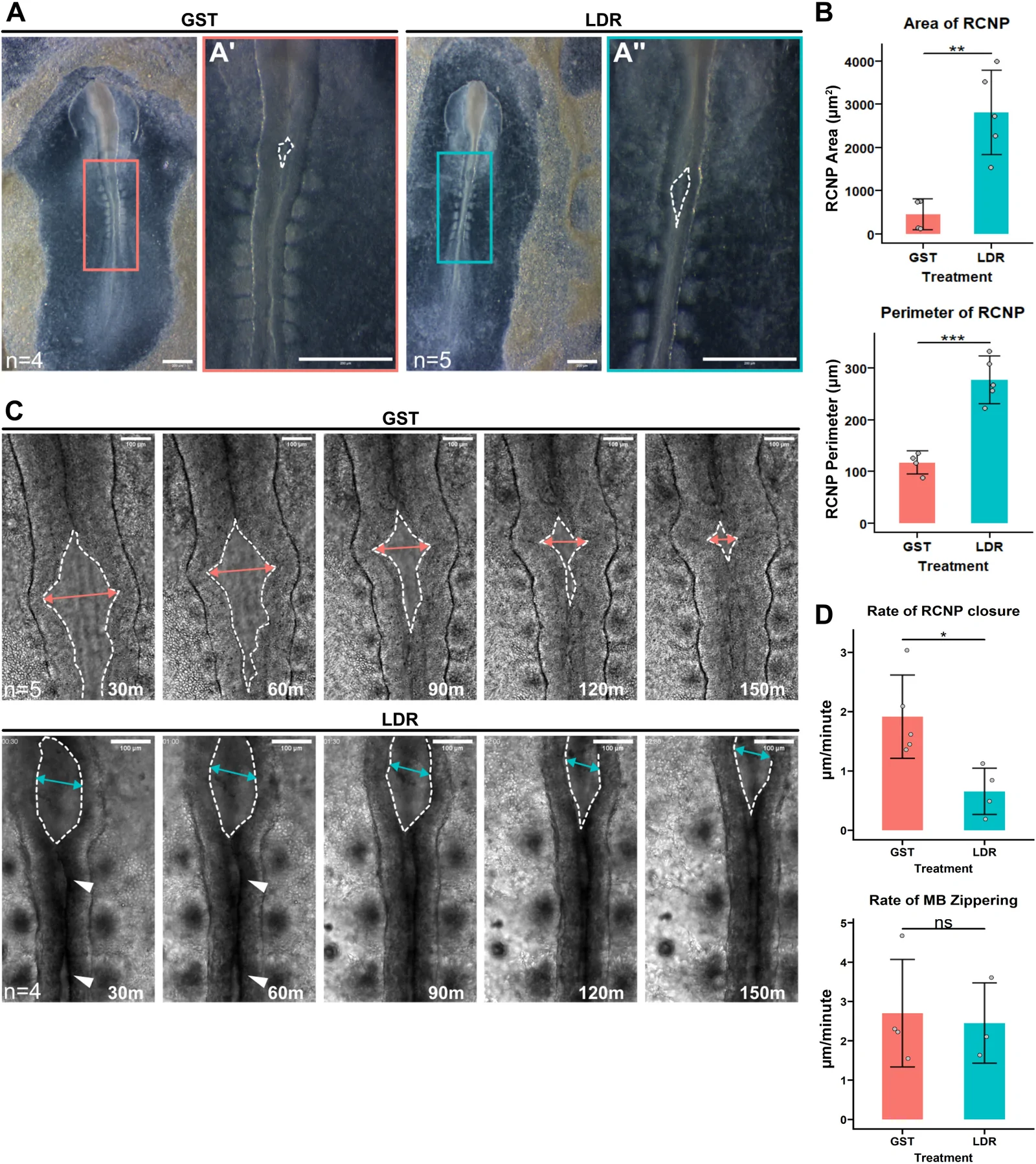
**CLDN3 depletion decreases the rate of neural fold movement during fusion.** (A-A″) Representative images of control (GST; *n*=4) and CLDN3-depleted (LDR; *n*=5) embryos at HH9. Coloured boxes indicate the regions shown at higher magnification on the right; white dashed lines outline the RCNP. (B) Quantification of RCNP area and perimeter. Coloured bars show mean; each point is from one embryo. Error bars show s.d. (C) maximum intensity projections of still images from live imaging of control (GST; *n*=5) (Movie 3) and CLDN3-depleted (LDR; *n*=4) (Movie 4) embryos taken at 30-min (m) intervals. White dashed lines outline the RCNP and double-headed arrows (GST: pink; LDR: blue) mark the measured distance between neural folds in the RCNP. White arrowheads point to open regions between neural folds in the spinal region. (D) Quantification of the rate of change of zippering in midbrain (MB) and quantification of the rate of change between fusing RCNP neural folds. Coloured bars (GST: pink; LDR: blue) show mean, and each point represents one embryo. Error bars show s.d. Statistical significance was evaluated with two-tailed, unpaired Student's *t*-test. ***P<0.005, **P<0.01, *P<0.05. ns, not significant (*P*>0.05). Scale bars: 200 μm (A); 100 μm (C).

To visualize the movement of the RCNP neural folds in the CLDN3-depleted embryos, we performed live imaging on chick embryos cultured on either LDR (400 μg/ml) or molarity-matched control GST (266 μg/ml) agar-albumen plates. All control embryos (*n*=5/5) completed neural tube closure during the 15 h that the embryos were observed with live imaging, while only half of the CLDN3-depleted embryos (*n*=2/4) had closed neural tubes at the end of imaging. We first compared cranial neural fold fusion in the midbrain region and determined that there was no effect on the rate of zippering in the CLDN3-depleted embryos relative to control embryos (GST: 2.7±1.4 μm/min; LDR: 2.5±1.0 μm/min; mean±s.d.; *P*=0.79) (Fig. 3C,D). Next, to compare the rate of narrowing of the RCNP as its neural folds moved together between control (Movie 3) and CLDN3-depleted (Movie 4) embryos, we determined the change in the distance between neural folds at the midpoint of the RCNP over time. On average, the rate of RCNP movement was slower in CLDN3-depleted embryos (GST: 1.9±0.7 μm/min; LDR: 0.6±0.3 μm/min; mean±s.d.; *P*=0.01) (Fig. 3C,D). These data suggest that the larger RCNP in CLDN3-depleted embryos was due to a decrease in the rate of neural fold movement medially, a process that occurs throughout buttoning. We predict that this delayed narrowing movement between button points contributes to the spinal NTDs we observed in CLDN3-depleted embryos (Legere et al., 2024).

### CLDN3 depletion increases the level of pMLC apical enrichment

In our previous analysis of CLDN3-depleted chick embryos, we traced the outline of cells from scanning electron micrographs and found that on average the apical cell areas of the CLDN3-depleted embryo were smaller and bicellular apical actin was decreased (Legere et al., 2024). To examine downstream effects of CLDN3 depletion that may underlie changes in apical cell morphology, we examined the expression of pMLC in the non-neural ectoderm of control (*n*=9) and CLDN3-depleted (*n*=14) embryos undergoing neural fold fusion (Fig. 4A). In contrast to the mediolateral cables of pMLC observed in the neural ectoderm (Nishimura et al., 2012), pMLC in the non-neural ectoderm is weakly localized throughout the cytoplasmic face of the membrane and in the cytoplasm. CLDN3 is expressed throughout all cells of the non-neural ectoderm but not in the neural ectoderm (Fig. S3D; Collins et al., 2013; Haworth et al., 2005) and we showed previously that LDR treatment depletes its localization at tight junctions throughout the non-neural ectoderm (Legere et al., 2024). As expected, no effect on pMLC intensity was observed in the neural ectoderm of CLDN3-depleted embryos (*n*=6) (GST: 2.2±0.9; LDR: 3.5±2.1; mean±s.e.m.; *n*=3; *P*=0.25) (Fig. S3C). However, on average the overall intensity of pMLC compared to the unchanging ZO1 expression (Fig. S3A) was increased in the non-neural ectoderm of CLDN3-depleted embryos (GST: 1±1.2; LDR: 2.2±1.7; mean±s.e.m.; *P*=0.04) (Fig. 4B,C) in both the membrane (GST: 0.8±0.9; LDR: 1.7±1.2; *P*=0.04) and cytoplasmic (GST: 1.3±1.6; LDR: 2.9±2.2; *P*=0.04) regions of the cells (Fig. 4C′; Fig. S3B). The increased level of pMLC in CLDN3-depleted embryos may partially explain the smaller apical cell surface areas previously measured in the non-neural ectoderm (Legere et al., 2024), since increased levels of pMLC are known to correlate with increased actomyosin constriction and decreased apical cell surface area (Kinoshita et al., 2008). In CLDN3-depleted embryos, we observed several instances of increased pMLC on some cells facing the open neural folds (Fig. 4B), that might be situations in which CLDN3 depletion increased the tension in the zone closest to the neural fold edge.

**Fig. 4.**
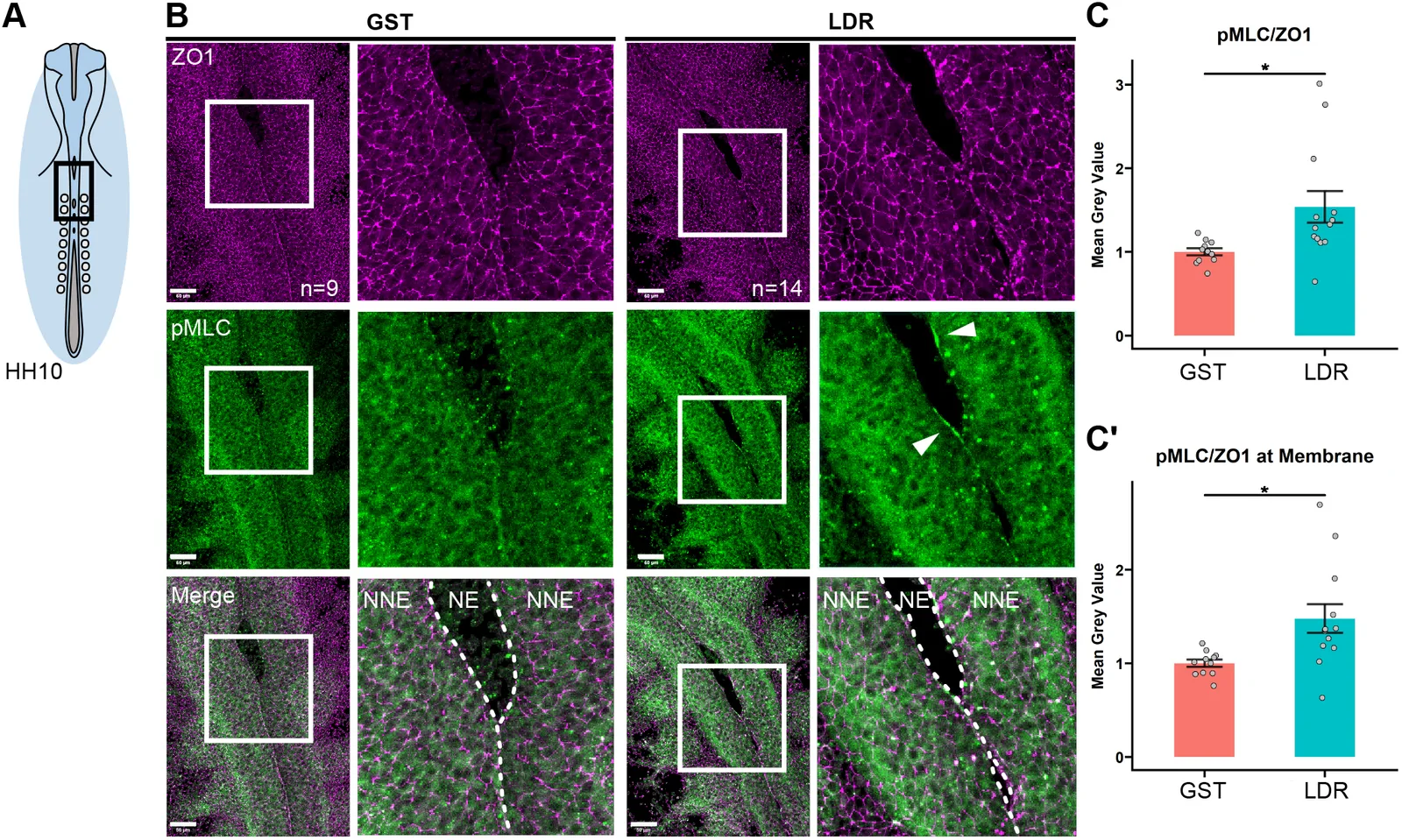
**CLDN3 depletion increases the abundance of pMLC.** (A) Schematic of an HH10 embryo with black box showing the approximate area imaged. (B) Representative ZO1 (magenta) and pMLC (green) immunofluorescence surface-projected images of control (GST; *n*=9) and CLDN3-depleted (LDR; *n*=14) embryos cultured for 15 h. White boxes indicate regions shown at higher magnification on the right. White dashed lines outline edges of neural folds on the merged images with non-neural ectoderm (NNE) and neural ectoderm (NE) labelled. White arrowheads point to areas of increased pMLC staining on the neural fold edge. (C,C′) Quantification of pMLC/ZO1 signal intensity normalized per experiment over the entire image (C) and at membrane regions defined by ZO1 signal (C′). Coloured bars [control (GST): pink; CLDN3 depleted (LDR): blue] show mean, and each point represents one embryo. Error bars show s.e.m. Statistical significance was evaluated with two-tailed unpaired Student's *t*-test. *P<0.05. Scale bars: 50 μm.

### CLDN3 depletion increases tension in the non-neural ectoderm during neural tube development

To determine whether CLDN3 depletion affected the tension in the non-neural ectoderm, we used a laser ablation (Hutson et al., 2003) approach that has been used to infer tissue tension perpendicular to the direction of the ablations during neural tube development in mouse (Marshall et al., 2022), *Xenopus* (Morita et al., 2012) and chick (Maniou et al., 2024). Chick embryos were cultured on GST- or LDR-containing agar-albumen plates until HH8 and then stained with CellMask prior to imaging. Laser ablation was performed along a 50 μm line. To infer tension of the epithelial layer, recoil was measured as the change in distance between two landmarks along the midline of the ablation before and after ablation. Higher recoil correlates with increased tension, while lower recoil correlates with lower tension (Liang et al., 2016). First, we assessed the amount of tension in the neural ectoderm and the non-neural ectoderm. Tension in the neural ectoderm is anisotropic, and mediolaterally (ML) directed cuts had higher recoil than anterior-posteriorly (AP) directed cuts in control embryos (*n*=9) (ML: 4.0±3.7 μm; AP: 0.8±0.7 μm; mean±s.d.; *P*=0.02). We did not observe a significant difference in recoil in either direction between control and CLDN3-depleted embryos in the neural ectoderm [GST ML (*n*=9): 4.0±3.7 μm; LDR ML (*n*=5): 4.5±5.9 μm; *P*=0.84; GST AP (*n*=10): 0.8±0.7 μm; LDR AP (*n*=5): 1.2±1.3 μm; *P*=0.47] (Fig. S4A). This was expected because CLDN3 is not expressed in the neural ectoderm. However, it also suggests that any changes in the non-neural ectoderm do not result in secondary effects on the neural ectoderm. In contrast, tension was not anisotropic in the non-neural ectoderm of control embryos (*n*=10) (ML: 3.7±3.7 μm; AP: 4.1±3.2 μm; mean±s.d.; *P*=0.65) or in the non-neural ectoderm of CLDN3-depleted embryos (*n*=8) (ML: 8.4±4.7 μm; AP: 8.4±3.8 μm; *P*=0.98). However, there was a significant difference in tension in the non-neural ectoderm between control (*n*=10) and CLDN3-depleted (*n*=8) embryos in both directions, with higher tension being observed in CLDN3-depleted embryos (GST ML: 3.7±3.7 μm; LDR ML: 8.4±4.7 μm; *P*=0.03; GST AP: 4.1±3.2 μm; LDR AP: 8.4±3.8 μm; *P*=0.02) (Fig. 5B). Because non-neural ectoderm tension was not anisotropic, we also compared the combined recoil measurements for both directions and confirmed that the recoil observed after ablation was significantly higher in CLDN3-depleted embryos than in control embryos (GST: 3.8±2.9 μm; LDR: 8.7±2.4 μm; *P*=0.001) (Fig. 5C,D).

**Fig. 5.**
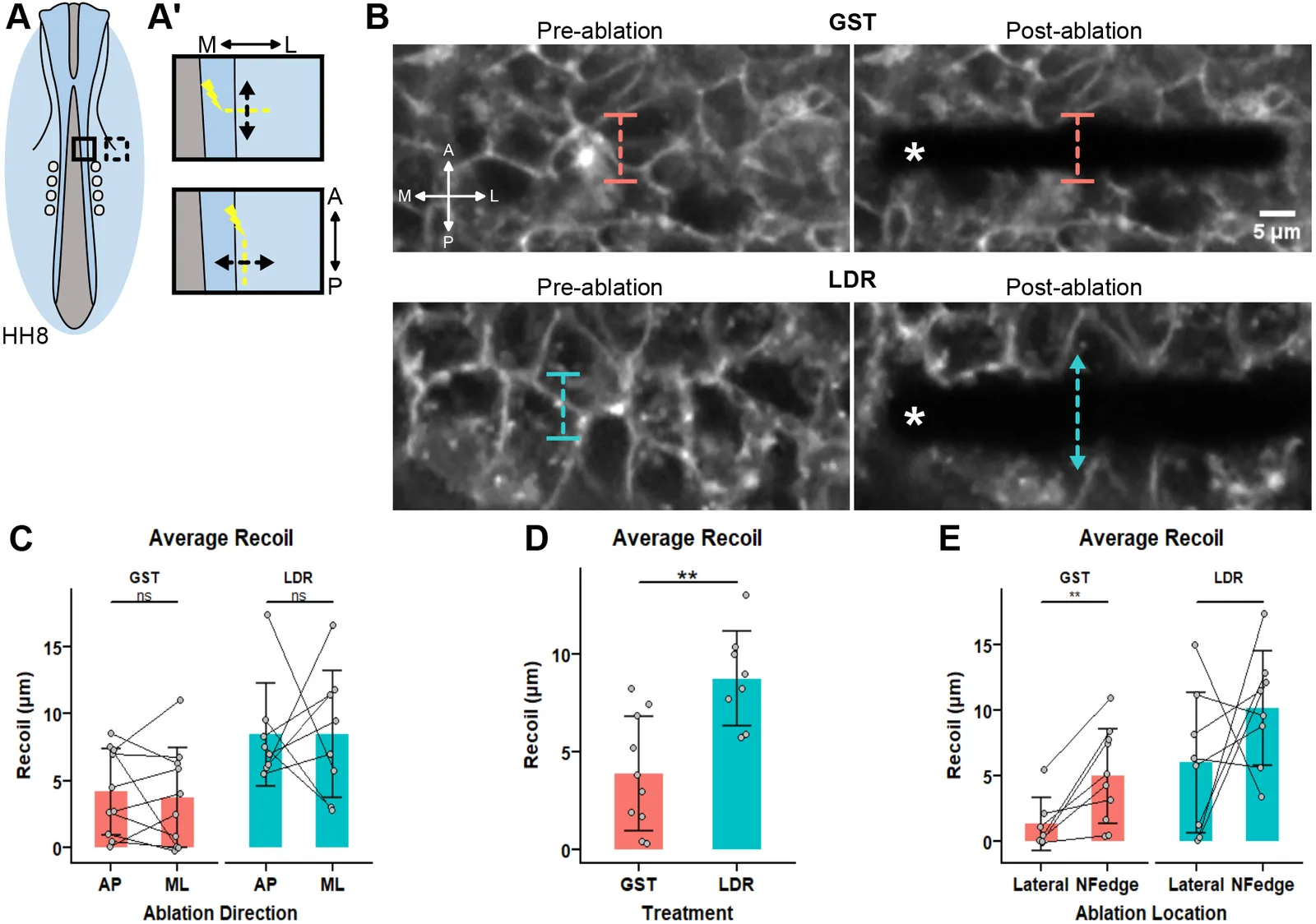
**CLDN3 depletion increases tension in the non-neural ectoderm during neural tube development.** Non-neural ectoderm tension was assessed with tissue-wide (approximately five to ten cell widths) laser ablations. Recoil perpendicular to the direction of ablation was measured by taking the difference in lengths between landmarks pre- and post-ablation. (A) Schematic of an HH8 embryo to illustrate approximate regions ablated on neural fold edge (black box) and lateral non-neural ectoderm tissue (dashed black box). (A′) Magnified view of the region in the black box showing examples of laser ablation (yellow lightning bolt with dashed line) and the direction of tissue recoil (dashed black arrows) in medial (M) to lateral (L) and anterior (A) to posterior (P) directions. (B) Representative single *z*-stack images of control (GST; *n*=10) and CLDN3-depleted (LDR; *n*=8) embryos; orientation is shown in the lower left corner, and images are labelled ‘pre-ablation’ and ‘post-ablation’ from left to right. Dashed brackets and double-headed arrow represent the lengths measured between landmarks on pre- and post-ablated images. Asterisks mark ablation. (C) AP or ML recoils were averaged per embryo, and two-tailed paired Student's *t*-tests used to assess difference in recoils in AP and ML directions in control (GST; *n*=10) and CLDN3-depleted (LDR; *n*=8) embryos. AP and ML ablation directions were performed on the same embryo, but on opposing neural folds. (D) Average recoil of both AP and ML ablations compared between control (GST; *n*=10) and CLDN3-depleted (LDR; *n*=8) embryos. Two-tailed unpaired Student's *t*-test was used. (E) Average recoil of AP and ML ablations in lateral non-neural ectoderm tissue (20 cell widths from neural fold edge) and ablations done within cells at neural fold (NF) edge. Two-tailed paired Student's *t*-test used to compare recoil within control (GST; *n*=7) and CLDN3-depleted (LDR; *n*=8) embryos. (C-E) All graphs show mean in bars (GST: pink; LDR: blue), errors bars show s.d., points represent average recoil (μm) per embryo, and lines connect measurements made on the same embryo. **P<0.01. ns, not significant (*P*>0.05). Scale bars: 5 μm.

To understand whether the increased tissue tension in CLDN3-depleted embryos is limited to specific regions or across the entire non-neural ectoderm, we compared recoils measured at the neural fold edge with recoils in more lateral regions ∼20 cells away from the edge (Fig. 5A,A′). In control embryos, the cells at the neural fold edge were under higher tension (*n*=7; neural fold edge: 4.9±3.6 μm; lateral: 1.3±1.9 μm; mean±s.d.; *P*=0.006), while in CLDN3-depleted embryos the tension in cells at the neural fold edge was not significantly higher than lateral cells (*n*=8; neural fold edge: 10.1±4.3 μm; lateral: 6.0±5.3 μm; *P*=0.24). In summary, the non-neural ectoderm in CLDN3-depleted embryos exhibited higher tension at both the neural fold edge and in more lateral non-neural ectoderm tissue than in control embryos (neural fold edge: *P*=0.01; lateral: *P*=0.04) (Fig. 5E). Thus, the increase in non-neural ectoderm tension in CLDN3-depleted embryos appears to be related to reduced levels of CLDN3 in tight junctions independent of their proximity to the midline.

### Treatment with the myosin II inhibitor blebbistatin rescued NTDs in CLDN3-depleted embryos

Given the increased pMLC levels (actomyosin contraction) and the increased tension in the non-neural ectoderm of CLDN3-depleted embryos, we wanted to determine whether reducing the tension in CLDN3-depleted embryos would reduce the incidence of NTDs. To do this, we used the myosin II inhibitor blebbistatin, which has previously been shown to reduce tension in the surface ectoderm of mouse embryos at a concentration that does not directly impair progression of neural tube closure (Nikolopoulou et al., 2019). Embryos were cultured on GST- or LDR-containing agar-albumen plates and treated with 2.5-50 μM blebbistatin at the beginning of neural fold fusion (∼HH8). We observed a dose-dependent effect of blebbistatin on neural tube development in control embryos (Fig. 6A,B; Fig. S5C). At the lowest dose tested, 2.5 μM blebbistatin, one-third of the control embryos displayed a mild cranial NTD (*n*=1/3), at 5 μM blebbistatin 100% of embryos had NTDs (*n*=8/8) that varied in severity and location, and at 50 μM blebbistatin 100% of embryos displayed NTDs in both cranial and spinal regions (*n*=3/3) and two-thirds of the embryos (*n*=2/3) were noticeably shorter along their anterior-posterior axes than untreated embryos (Fig. 6A,B). The increased incidence of NTDs in embryos cultured in the intermediate dose of 5 μM blebbistatin (100%) versus control media (25% NTDs; *n*=12) was also significant (Fisher's Exact Test, *P*=0.0014). The 5 μM blebbistatin dose caused NTDs most similar in penetrance and severity to the NTDs caused by CLDN3 depletion (94% NTDs, *n*=17; Fisher's Exact Test to GST, *P*=0.0002) (Fig. 6A,C; Fig. S5C). When combined with CLDN3 depletion, the lowest and highest doses of blebbistatin had no effect on the incidence of NTDs compared to CLDN3 depletion alone (400 μg/ml LDR: 94% NTDs, *n*=16/17; LDR+2.5 μM blebbistatin: 100% NTDs, *n*=3/3; LDR +50 μM blebbistatin: 92% NTDs, *n*=11/12) (Fig. 6A,C). However, there was a significant decrease in the number of embryos with NTDs when CLDN3-depleted embryos were treated with 5 μM blebbistatin (LDR+5 μM blebbistatin: 36% NTDs, *n*=5/14; Fisher's Exact Test, *P*=0.0012) (Fig. 6A,C; Fig. S5B,C). The decrease in the number of embryos with NTDs when 5 μM blebbistatin was combined with LDR was also significant compared to treatment with 5 μM blebbistatin alone (Fisher's Exact Test, *P*=0.005). These data suggest that the CLDN3-depleted NTDs are caused by increased actomyosin contraction and that the phenotype can be rescued when actomyosin contraction is reduced with the myosin II inhibitor blebbistatin.

**Fig. 6.**
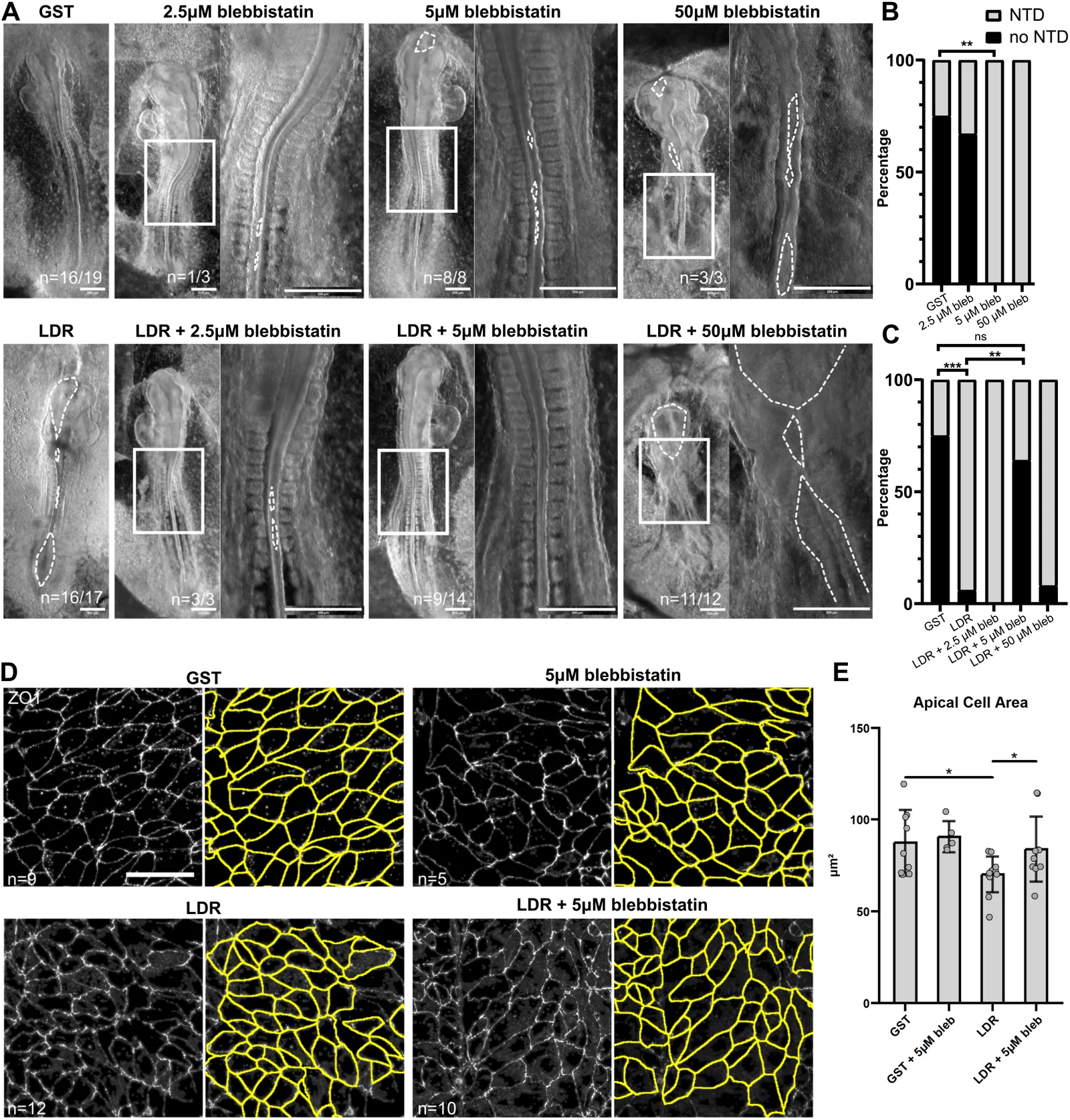
**Treatment with the myosin II inhibitor blebbistatin rescues the NTDs in CLDN3-depleted embryos.** (A) Representative images of GST+DMSO control (*n*=16/19) and 400 μg/ml LDR+5 μM blebbistatin (*n*=9/14) without NTDs, 2.5 μM blebbistatin (*n*=1/3), 5 μM blebbistatin (*n*=8/8), 50 μM blebbistatin (*n*=3/3), 400 μg/ml LDR+DMSO (*n*=16/17), 400 μg/ml LDR+2.5 μM blebbistatin (*n*=3/3) and 400 μg/ml LDR+50 μM blebbistatin (*n*=11/12) treated embryos with NTDs after 24 h of culture. Embryos were treated with blebbistatin at HH8. White boxes mark regions shown at higher magnification; white dashed lines outline open NTDs. (B,C) Quantification of the percentage of embryos with NTDs (black: no NTD; grey: NTD). Fisher's Exact Test was used to compare the proportion of NTDs between treatments. (D) Representative ZO1 (white) immunofluorescence surface-projected images of GST (GST+DMSO; *n*=9), 5 μM blebbistatin (GST+5 μM blebbistatin; *n*=5), LDR (LDR+DMSO; *n*=12), and LDR+5 μM blebbistatin (*n*=10) at the level of the first somite. Yellow lines outline apical cell areas measured after segmentation of ZO1 signal on surface-projected images. (E) Quantification of average apical cell area (μm^2^) per embryo. Grey bars show the mean, dark grey dots mark average apical cell areas of individual embryos, and error bars show s.d. Statistical significance was measured between GST and LDR, and LDR and LDR+5 μM blebbistatin with two-tailed, unpaired Student's *t*-test. *P<0.05, **P<0.01, ***P<0.001. ns, not significant (*P*>0.05). Scale bars: 200 μm (A); 25 μm (E).

To determine whether the ability of 5 μM blebbistatin to rescue NTDs caused by CLDN3 depletion was upstream or downstream of the increased levels of pMLC observed in CLDN3-depleted embryos (Fig. 4), we assessed pMLC expression in control embryos, control embryos treated with 5 μM blebbistatin, and CLDN3-depleted embryos treated with 5 μM blebbistatin (Fig. S5A,A′). The pMLC/ZO1 ratio in the non-neural ectoderm of 5 μM blebbistatin-treated embryos (*n*=5) was unchanged compared to control embryos (*n*=6) (GST+DMSO: 0.47±0.09; 5 μM blebbistatin: 0.45±0.08; mean±s.d.; *P*>0.05). Similarly to CLDN3-depleted embryos, there was a significant increase in pMLC immunofluorescence intensity in the non-neural ectoderm of 5 μM blebbistatin+CLDN3-depleted embryos (*n*=3) compared to embryos treated with only 5 μM blebbistatin (LDR+5 μM blebbistatin: 1.32±0.34; 5 μM blebbistatin: 0.45±0.08; *P*=0.04). There was also no change in the localization of pMLC in blebbistatin-treated embryos as assessed by pMLC/ZO1 membrane-to-cytoplasmic signal (DMSO: 2.18±0.33; 5 μM blebbistatin: 2.05±0.44; *P*=0.69) or Pearson's R colocalization between pMLC and ZO1 (DMSO: 0.37±0.05; 5 μM blebbistatin: 0.39±0.11; *P*=0.76). Therefore, the rescue of CLDN3-depleted NTDs by blebbistatin does not appear to be acting by reducing the levels of pMLC and is most likely acting downstream of this effect or via a parallel pathway due to release of excess non-neural ectodermal tension/actomyosin contraction through inhibition of myosin II. This would be predicted because blebbistatin is known to act directly on myosin II (Allingham et al., 2005) with contradictory effects on pMLC that may be context dependent (Berger et al., 2018; Escuin et al., 2015; Hirano and Hirano, 2016; Kovács et al., 2004).

Treating CLDN3-depleted embryos with 5 μM blebbistatin also rescued the previously reported smaller apical cell areas (Legere et al., 2024). ZO1 immunofluorescence images were surface-projected and segmented to measure apical cell areas of the non-neural ectoderm on neural folds in HH8-HH9 staged embryos. We repeated our previous finding that average non-neural ectodermal apical cell surface areas were smaller in CLDN3-depleted embryos (*n*=12) compared to control embryos (*n*=9) (GST+DMSO: 87±18 μm^2^; LDR+DMSO: 70±10 μm^2^; mean±s.d.; *P*=0.01) (Fig. 6D,E). Treating CLDN3-depleted embryos with 5 μM blebbistatin (*n*=10) increased average apical cell areas to baseline (LDR+DMSO: 70±10 μm^2^; LDR+5 μM blebbistatin: 84±18 μm^2^; *P*=0.032) (Fig. 6D,E). These data provide further evidence that the change in apical cell morphology, hypothesized to be due to increased tension/actomyosin contraction, may underlie the formation of NTDs in CLDN3-depleted embryos.

## DISCUSSION

CLDN3-depleted chick embryos provide an excellent model in which to investigate the specific role of the non-neural ectoderm in neural fold fusion. Our live-imaging analyses confirmed previous analyses of static images that the spinal neural folds of chick embryos close by buttoning rather than the zippering activity observed in mouse and at the cranial and most-posterior ends of chick embryos (van Straaten et al., 1993). Button-like closure is also observed in *Drosophila* dorsal vessel closure (Swope et al., 2014), small regions of the mouse midbrain (Wang et al., 2017) and hindbrain (Pyrgaki et al., 2010), as well in the spinal region of pig (van Straaten et al., 2000) and rabbit (Peeters et al., 1998b) embryos during neural fold fusion. Our data support that CLDN3 is essential for the progression of spinal buttoning during neural fold fusion by regulating non-neural ectodermal tension through pMLC activity (Fig. 7). Our data suggest that the buttoning process of neural fold fusion, unlike zippering, is more sensitive to changes in non-neural ectodermal tension during neural fold convergence. Our work contributes to the broader understanding about functions of the claudin family of tight junction protein in regulating the cytoskeleton to mediate tension, as well as the crucial role of the non-neural ectoderm in neural fold fusion.

**Fig. 7.**
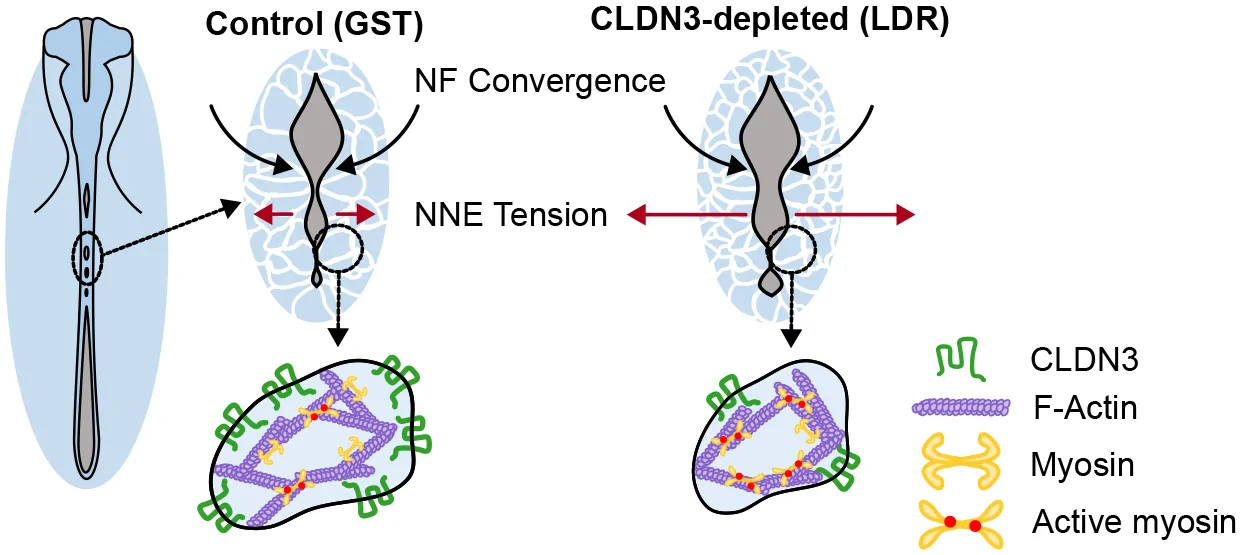
**Model of the role of CLDN3 in the non-neural ectoderm during neural fold fusion**. Schematic of an HH10 chick embryo undergoing neural fold fusion. Dashed circles indicate regions shown at higher magnification. Cell outlines are shown with white lines and convergence of neural folds with black arrows. The underlying tension in the non-neural ectoderm (NNE) is shown with red arrows. An apical view of NNE cells showing CLDN3 at the membrane with apical actin cortex formed by F-actin and myosin is shown beneath. Active myosin is shown as unfolded, bipolar filaments with phosphorylation of myosin light chain (pMLC) indicated with red dots. The tension in the NNE is increased in CLDN3-depleted embryos, shown by longer red arrows. Increased actomyosin contraction, through increased pMLC, is shown in an apical view of CLDN3-depleted cells with smaller apical areas and increased pMLC. CLDN3, through regulation of pMLC and actomyosin contraction, maintains the balance of tissue tension in the NNE to mediate neural fold fusion. CLDN3: green; actin cortex: purple; pMLC: yellow with red dot (phosphorylation); non-neural ectoderm: blue; neural ectoderm: grey.

Previous studies investigating the biomechanics of chick neural tube development showed that the dorsal neural ectodermal tissue becomes stiffer during neural fold elevation and fusion, suggesting changes to biophysical forces (Handler et al., 2023; Zhang et al., 2019). Indeed, the neural ectoderm within the cranial region generates force through bending of the dorsolateral hinge points during chick neural fold fusion (Roellig et al., 2022). Generation of medially directed force within the chick neural ectoderm was also observed at the level of the RCNP (Maniou et al., 2024). Additionally, a pulling force from the elongation of the neural tube and notochord along the anterior-posterior axis drives zippering in the PNP (Xiong et al., 2020). Our laser ablation study in the neural ectoderm of the PNP supports this work as we determined that the neural ectoderm has higher tension along the anterior-posterior axis as measured by recoil from mediolaterally directed ablations (Fig. 3A). Additionally, our microsurgical experiments showed that unlike the cranial or mid-spine cuts, cuts in the PNP did not ‘heal’ and instead expanded along the anterior-posterior axis, suggesting greater force within that region (Fig. 2).

In contrast to the neural ectoderm, the non-neural ectoderm did not exhibit tension anisotropy, and it was less tense than the neural ectoderm, suggesting that the non-neural ectoderm is passive to forces generated by the neural ectoderm. However, previous microsurgical experiments show that the non-neural ectoderm is required for neural fold fusion (Alvarez and Schoenwolf, 1992), specifically the medial region of the non-neural ectoderm that is part of the neural folds (Hackett et al., 1997). Further evidence for the importance of tissue tension in neural tube closure is that preventing a loss of tension in the extra-embryonic and vitelline membrane in chick embryos causes defects in elongation and neural tube development in chick embryos (Kunz et al., 2023). The NTDs in CLDN3-depleted embryos were associated with increased tension in the non-neural ectoderm. Similarly, increasing non-neural ectoderm tension in *Xenopus* also causes NTDs (Christodoulou and Skourides, 2022). Also, like our observation of NTDs in embryos treated with blebbistatin, which is known to decrease tissue tension (Nikolopoulou et al., 2019), others have shown that decreasing tissue tension causes NTDs in chick (Kunz et al., 2023) and *Xenopus* (Christodoulou and Skourides, 2022). Based on our data and the work of others, we hypothesize that a precise balancing of epithelial tissue tension in the non-neural ectoderm is required to permit neural fold fusion during buttoning. Too much tension, as observed in the CLDN3-depleted embryos, may prevent the medial movement of the tissue, which is required for the neural folds to simultaneously make contact along the length of the neural tube. In contrast, too little tension, as observed in blebbistatin-treated embryos, also impacted the ability of the neural folds to maintain contact during closure (Fig. 6A,B). Maintaining this balance of tension in the non-neural ectoderm may be particularly important during buttoning as there is no additional force being generated by an actomyosin cable and/or a zippering point that could act to pull the neural folds together to maintain contact (Maniou et al., 2021; Molè et al., 2020).

We are not the first to suggest a relationship between tight junctions and regulation of the cytoskeleton and tissue tension. Protein interactions between claudins and apical polarity complexes may result in altered cytoskeletal rearrangements and/or changes to cell contractility. Previously, we observed altered localization of PAR3 and MPP5 within the apical domain of CLDN3-depleted cells (Legere et al., 2024). Apical polarity complexes are also important mediators of the cytoskeleton and cell contractility (Nance and Zallen, 2011; Osswald et al., 2022) and further work is required to understand the mechanistic relationship between claudins, apical polarity complexes and the cytoskeleton. Additionally, tight junction components are known to interact with RhoGTPases or regulatory proteins (Chumki et al., 2022; Haas et al., 2024; Itoh, 2013; Pinto-Dueñas et al., 2024; Suarez-Artiles et al., 2022; Tambrin et al., 2025) and simultaneous depletion of CLDN4 and CLDN8 in the neural ectoderm of chick embryos results in decreased RHOA, CDC42 and pMLC at the apical lateral membrane (Baumholtz et al., 2017). In contrast, RHOA and CDC42 are not decreased in the non-neural ectoderm in CLDN3-depleted embryos (Legere et al., 2024), and instead we found that pMLC is increased in the non-neural ectoderm. These data suggest multiple functions for claudin family members in cytoskeletal regulation. Claudin ‘quinKO’ Madin–Darby canine kidney MDCK cells in which *CLDN1*, *CLDN2*, *CLDN3*, *CLDN4* and *CLDN7* are deleted have altered actomyosin organization with highly developed circumferential actin bundles and increased pMLC surrounding the cell junctions (Otani et al., 2019). Overexpression of CLDN3 in the quinKO cells reduces the circumferential actin staining (Fujiwara et al., 2022). Claudin quinKO MDCK cells also have increased myosin II accumulation at the cell junction and increased cortical tension during lumen expansion of MDCK cysts (Mukenhirn et al., 2024). Tight junctions may play a role in regulating resistance to mechanical stress (Nguyen et al., 2024) and/or tissue fluidity (Skamrahl et al., 2021), both functions that require a tight control of biomechanical sensing and response. Given these data and our own, the increase in actomyosin contraction and tissue tension observed in CLDN3-depleted embryos highlights an important function for CLDN3 in this process. Our data suggest that CLDN3 has the ability to act as a stabilizer that regulates and/or maintains the appropriate level of tension across the non-neural ectoderm that is permissive to neural fold fusion.

Our work aligns with the NTD phenotypes observed in mice with altered expression of the transcription factors *Grhl2* (Nikolopoulou et al., 2019; Ray and Niswander, 2016) and *Grhl3* (Ting et al., 2003). Both GRHL2 and GRHL3 are expressed in the non-neural ectoderm and known to regulate expression of multiple claudins, including CLDN3, and other cell junctional proteins in mice (Nikolopoulou et al., 2019; Ray and Niswander, 2016) and *in vitro* studies (Kimura-Yoshida et al., 2015; Senga et al., 2012). Both GRHL2 and GRHL3 regulate actomyosin dynamics in the non-neural ectoderm (Jaffe and Niswander, 2021; Nikolopoulou et al., 2019). Of particular relevance to our study, is the observation that loss of *Grhl2* expression leads to loss of CLDN3, and phenotypes similar to those we observed, including disorganized F-actin (Legere et al., 2024), over-accumulation of pMLC, and increased tension in the non-neural ectoderm (Marshall et al., 2023; Nikolopoulou et al., 2019). The parallels between the *Grhl2* null mouse model and our CLDN3-depleted embryo suggest that the changes to the cytoskeleton and increased non-neural ectodermal tension in the *Grhl2* null mice are mediated by the loss of CLDN3. The absence of NTDs in the *Cldn3* knockout mouse is predicted to be due to the presence of other claudin family members that are also regulated by GRHL2 but that are not present in the chick genome (Kerr et al., 2015). The ability of blebbistatin to partially rescue NTDs caused by RhoA inhibition or observed in *Zic2^ku/ku^* and *Grhl2^Axd/Axd^* mutant mice indicates that altered cytoskeletal dynamics are a major mechanism for the formation of NTDs (Escuin et al., 2023, 2015; Nikolopoulou et al., 2019). Variants in *Grhl3* (Kousa et al., 2019; Yang et al., 2019), claudins (including CLDN3) (Baumholtz et al., 2020) and cytoskeletal regulatory proteins (Au et al., 2021; Zou et al., 2020) have been identified in human populations with NTDs. Future pre-clinical studies are required to determine whether these proteins are therapeutic targets for the prevention of spinal NTDs.

## MATERIALS AND METHODS

### Chick embryo collection and culture

Chick embryos were collected from fertilized Lohmann (derived from White Leghorn) chick eggs (Ferme Gms, Saint-Roch-de-l'Achigan) incubated at 38.5°C until the desired stage following the HH staging guide (Hamburger and Hamilton, 1951). All embryos were handled according to the Canadian Council of Animal Care guidelines.

Chick embryos were cultured with filter papers on agar-albumen dishes following standard protocols (Chapman et al., 2001); for some experiments included in Fig. 6A,B, embryos were cultured using the Cornish pasty method (Nagai et al., 2011). For assessment of neural tube development, embryos were incubated at 38.5°C for ∼24 h until gastrulation stage HH4 and cultured for another ∼24 h until ∼HH12 when most of the neural tube development was complete. To deplete CLDN3 from tight junctions, HH4 embryos were treated with GST-tagged C-CPE variant C-CPE^LDR^ (LDR) (400 μg/ml) (Sonoda et al., 1999; Veshnyakova et al., 2012). The control treatment was molarity-matched GST (266 μg/ml). Blebbistatin (Sigma-Aldrich, B0560) treatment (*N*_experiments_=8) was carried out after ∼8 h of culture when embryos were HH7-HH8 (start of neural fold fusion). Blebbistatin solutions were made with dimethyl sulfoxide (DMSO). Control embryos received an equal volume of DMSO used in 5 μM blebbistatin. Embryos were imaged using a Zeiss Stereomicroscope Discovery V8. Statistical significance of the proportion of NTDs was tested with Fisher's Exact Test.

### Chick embryo microsurgeries

Chick embryos were sandwiched between two filter papers and cultured on agar-albumen dishes ventral side down until reaching the required stage. The vitelline membrane was peeled back over the position of microsurgery, or over an equivalent position on non-surgically treated controls (*N*_experiments_=6). Using an eyebrow hair as a knife, a piece of neural tube and some extra-embryonic tissue approximately the width of one somite were removed from the embryo at the specified positions. Surgeries were performed at HH8 to cut the embryo at the first neural fold contact point, at ∼HH9+ to cut the embryo during late neural fold fusion in the mid-spinal region, or at ∼HH11 to cut the embryo at the PNP of the embryos. Once cut, the embryos were incubated at 38.5°C until they reached HH12 or HH14 and assessed for spinal neural tube closure or PNP closure, respectively.

### Chick embryo live imaging

Chick embryos were incubated at 38.5°C for ∼24 h and collected on filter papers at HH4 and cultured on agar-albumen dishes containing either LDR (400 μg/ml) or molarity-matched GST (266 μg/ml) until the start of neural fold fusion (∼HH8). At ∼HH8, embryos were moved to a glass-bottom imaging dish (MatTek, P35G-1.5-10-C) coated with a thin (500 μl) agar-albumen layer with either LDR (400 μg/ml) or molarity-matched GST (266 μg/ml). Simple saline was dropped on the corners of the filter paper and the embryo was placed on a humidified platform and live imaged with a Zeiss Axio Observer A1 at 38.5°C. Embryos were imaged (*N*_experiments_=6) with an AxioCam MR R3 camera for up to 8 h with transmitted light halogen lamp (∼2.5 V) and minimal exposure time (100-200 msec) at 5- or 15-min intervals and *z*-stacks set up to centre on neural fold edges with ∼5 μm steps. Embryos were imaged with either Fluor 10×/0.5 M27 or Plan-Apochromat 20×/0.8 objectives. Maximum intensity projections were made of the videos and images were automatically adjusted for brightness and contrast with ImageJ. Landmarks at the centre of the RCNP were used for measuring the length between the fusing neural folds and bounding rectangles were used to compare widths between embryos. To measure the rate of zippering in the midbrain region of the embryo, the distance between the zippering point and the left somite was measured. Statistical significance was tested with two-tailed, unpaired Student's *t*-test.

### Chick embryo immunofluorescence

Fertilized eggs were incubated at 38.5°C for ∼24 h, and embryos were collected on filter papers at HH4 and cultured on agar-albumen dishes on either LDR (400 μg/ml) or molarity-matched GST (266 μg/ml) for 12 h (∼HH9). In experiments testing the effect of 5 μM blebbistatin (*N*_experiments_=2), blebbistatin was added at stage ∼HH8 and embryos were cultured until reaching the appropriate stage (HH8-HH9). Embryos were collected and fixed in the appropriate fixative for 1 h at room temperature [4% paraformaldehyde for ZO1, F-actin (phalloidin); 10% trichloroacetic acid for pMLC, ZO1, CLDN3]. For pMLC immunofluorescence experiments (*N*_experiments_=3), embryos underwent antigen retrieval with 10 mM sodium citrate for 25 mins at 100°C. After PBS washes, embryos were blocked in 10% normal goat serum with 0.3% Triton X-100 in PBS. Embryos were incubated with primary antibodies (ZO1: Invitrogen, 33--9100, 1:200; CLDN3: Abcam, ab15102, 1:50; pMLC: Cell Signaling Technology, 3674S, 1:50) overnight at 4°C. After PBS washes, embryos were incubated with secondary antibodies for 1 h at room temperature, and, if required, phalloidin (Invitrogen, A22287, 1:250) and/or DAPI (Thermo Fisher Scientific, D1306, 1:1000) was added in this step. Embryos were then washed with PBS and placed on microscope slides with FluorSave (Millipore, 34578920ML). Embryos were imaged with an LSM 880 Elyra AxioObserver with either a Plan-Apochromat 20×/0.8 M27 or Plan-Apochromat 63×/1.4 Oil DIC M27 objectives.

Immunofluorescence experiments performed on cryosections were conducted in a similar method as described above. Embryos were cryoembedded by incubating for 1 h in 5% sucrose, overnight at 4°C in 30% sucrose, 1 h in 50:50 30% sucrose:Cryomatrix and then flash-frozen in embedding moulds filled with Cryomatrix (Epredia, 67-690-06). Embryos were sectioned at 10 μm thickness using a Leica CM3030 cryostat.

### Analysis of immunofluorescence images

Images were analysed using ImageJ (Schindelin et al., 2012) and surface projections were made with the macro SurfacePeeler (github.com/DaleMoulding/SurfacePeeler) with the following settings: gaussian blur=2, default threshold, −2 to 2 (∼2 μm) pixel thickness. Thickness was set to capture the apical surface of non-neural ectoderm using ZO1 signal on orthogonal views. Analysis was primarily conducted on non-neural ectoderm, distinguished from the neural ectoderm by larger apical cell areas. Mean grey intensity was measured and the ratio of pMLC to ZO1 mean grey value was calculated. One embryo was removed from further analysis after outlier analysis. ZO1 signal was used to define cell membrane regions and mean grey intensity was measured within the membrane region and the inverse (cytoplasm) as previously described (Legere et al., 2024). For graphical representations, the average pMLC/ZO1 ratio per experiment was used as a normalization factor; however, statistical analysis (two-tailed, unpaired Student's *t*-test) was performed on raw data averaged per embryo. ZO1 immunofluorescence surface-projected images were segmented with the Cellpose Cyto3 model with manual segmentation error correction and apical cell areas were measured (*N*_experiments_=3) (Stringer and Pachitariu, 2025). Measurements were averaged across embryos and two-tailed, paired Student's *t*-tests were used to assess significance.

### Laser ablations

Laser ablations were performed at University College London (*N*_experiments_=4). Deklab fertilized chicken eggs were incubated at 38.5°C for ∼24 h and collected on filter papers at stage HH4. Embryos were cultured at 38.5°C on either LDR (400 μg/ml) or molarity-matched GST (266 μg/ml) until the start of neural fold fusion (∼HH8). Embryos were removed from the incubator immediately prior to imaging, flipped dorsal side up and the vitelline membrane over the area to be ablated/imaged was removed using a sharpened tungsten needle. Embryos were rinsed with simple saline then ∼20 µl of CellMask 647 (Invitrogen, C10046, 1:100) was added dropwise to the neural tube and incubated for 15 min in 38.5°C. CellMask was washed three times with PBS and placed into the 38.5°C incubator of an LSM880 Examiner. Embryos were imaged using a 20× NA:0.7 C Epiplan-Aprochromat objective (1024×1024, 9.63 pixels/μm) and 500-pixel long lines (∼50 μm) were ablated with a SpectraPhysics Mai Tai eHP DeepSee multiphoton laser (710 nm λ, 100% laser power). Images were captured immediately prior to and after ablation (0.96 s per image).

### Analysis of laser ablations

Final analysis was restricted to images of embryos up to stage HH10 without air bubbles (GST: *n*=10 embryos, 53 images; LDR: *n*=8 embryos, 62 images). Operators were unaware of treatment groups during analysis. Images were analysed using ImageJ (Schindelin et al., 2012) by selecting out the region around the ablation, running the plug-in pureDenoise with default parameters (Luisier et al., 2010), running StackReg with rigid body correction (EPFL) and selecting out the time points immediately before and after ablation. Landmarks that appeared in both pre- and post-ablation images on either side of the centre part of the ablation were selected and distances were measured three times and averaged. A bounding rectangle of the distance was used to measure the height of recoil and the difference between heights of pre- and post-ablation was measured to obtain the final recoil value. Measurements were averaged across embryos and Student's *t*-tests or paired Student's *t*-tests when applicable were used to assess significance.

## Supplementary Material



10.1242/joces.264758_sup1 Supplementary information
