# Supplementary material for: Increased tissue tension caused by depletion of CLDN3 in the non-neural ectoderm causes neural tube defects in chick embryos
Source: J Cell Sci. 2026 Sep 25;139(18):jcs264758. doi: 10.1242/jcs.264758 (PMC13680966; doi:10.1242/jcs.264758)
Supplement: Supplementary file 1 [file joces-139-264758-s1.pdf]

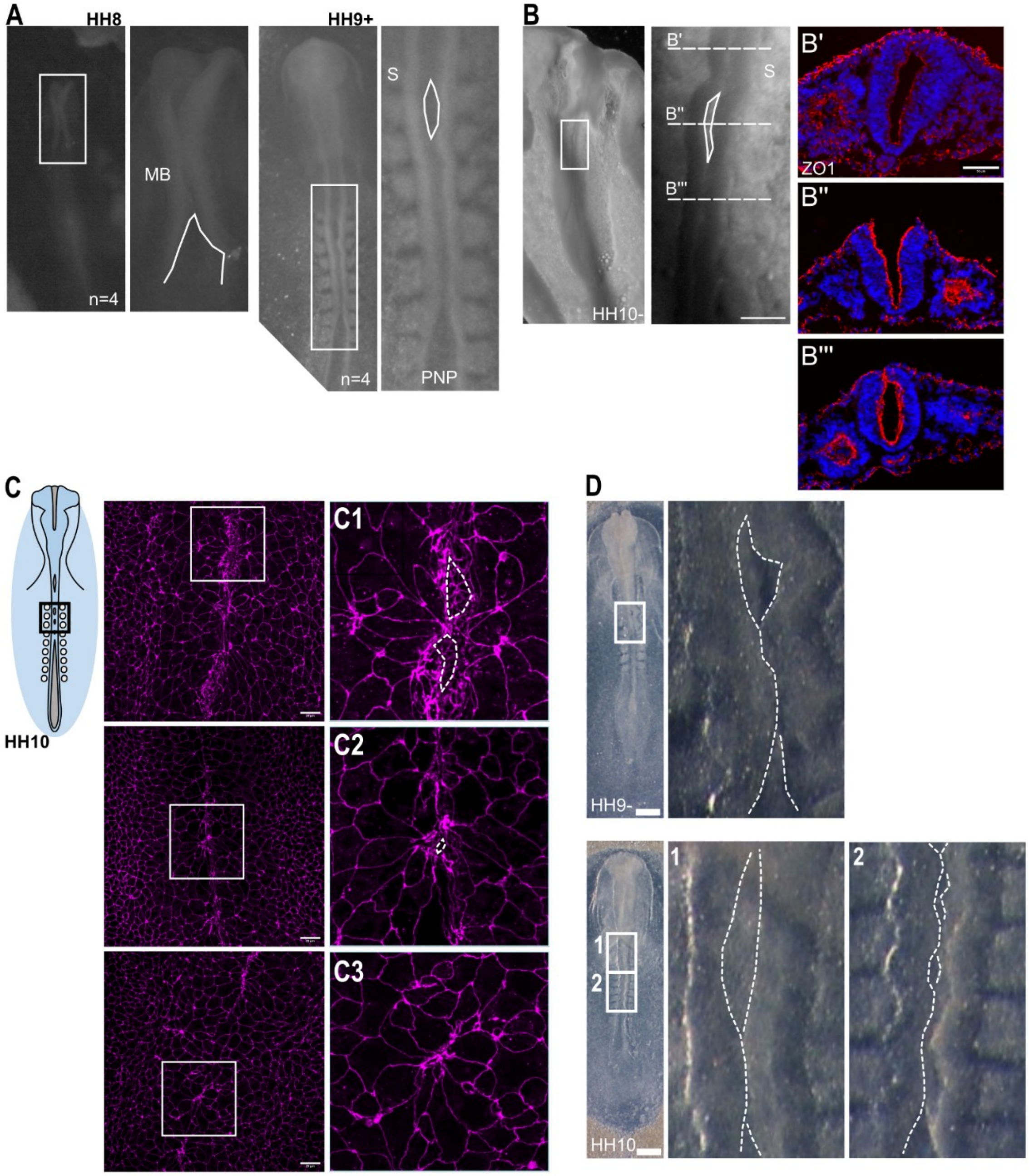

Fig. S1. Buttoning points are seen in *in ovo* and in *ex ovo* cultured embryos.

A) Representative images of *in ovo* embryos at HH8 (n=4) and HH9-HH10 (n=4). Open neural folds (NFs) along button gap are outlined with white lines. White boxes indicate regions shown in adjacent higher magnification images. MB: midbrain, S: somite, PNP: posterior neuropore. B) Dorsal view of a HH10- embryo. The white box indicates the region shown at higher magnification in the adjacent image. White lines outline the RCNP. White dashed lines show approximate locations of transverse sections in B', B'', and B'''. B'-B''') ZO1 immunofluorescence (red) on cryosections from a HH10- embryo (DAPI: blue) (n=3). Sections are ~100µm apart. C) Maximum intensity projections of representative images of ZO1 immunofluorescence (magenta) in HH10 (n=3) embryos. White boxes indicate regions shown in higher magnification images on right and white dotted lines outline gaps between NFs. C1) open gaps, C2) fusing gaps, and C3) fused gap between NFs. D) Representative brightfield images of fixed stage HH9 (n=17) and HH10 (n=12). White boxes indicate regions shown at higher magnification in images on the right. White dotted lines drawn on the edges of the NFs. Scale bars: B) 100 µm, B') 50 µm, C)20 µm, D) 200 µm.

Journal of Cell Science • Supplementary information

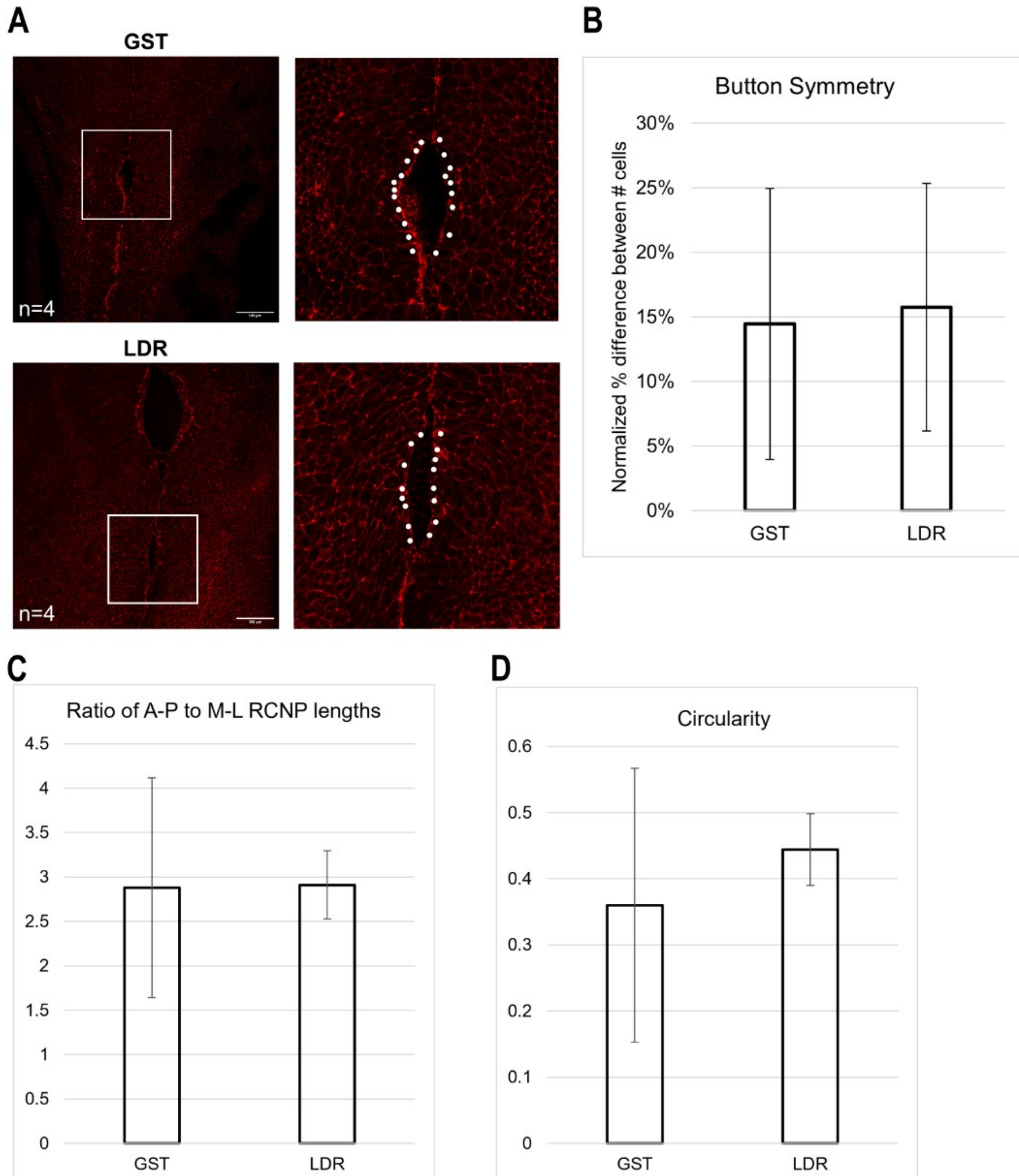

Fig. S2. Analysis of button symmetry in CLDN3-depleted embryos

A) Representative maximum intensity projection images of ZO1 immunofluorescence (red) of Control (GST; n=4) and CLDN3-depleted (LDR; n=4). White squares indicate region shown at higher magnification on right and white dots denote cell count on either side of the buttons. B) Difference between the number of cells on the left and right side of the buttons (normalized by average number of cells on left and right side) as percentages in control (GST; n=4) and CLDN3-depleted (LDR; n=4) embryos. C) Ratio of Anterior-Posterior (AP) to Medial-Lateral (ML) lengths of the rhombocervical neuropore (RCNP) and D) Circularity ($4\pi*area/perimeter^2$) of RCNPs between control (GST; n=4) and CLDN3-depleted (LDR; n=5). Bars show the mean and error bars show standard deviation. Statistical significance was evaluated with Student's T-test. Scale bar: A) 100 µm.

Journal of Cell Science • Supplementary information

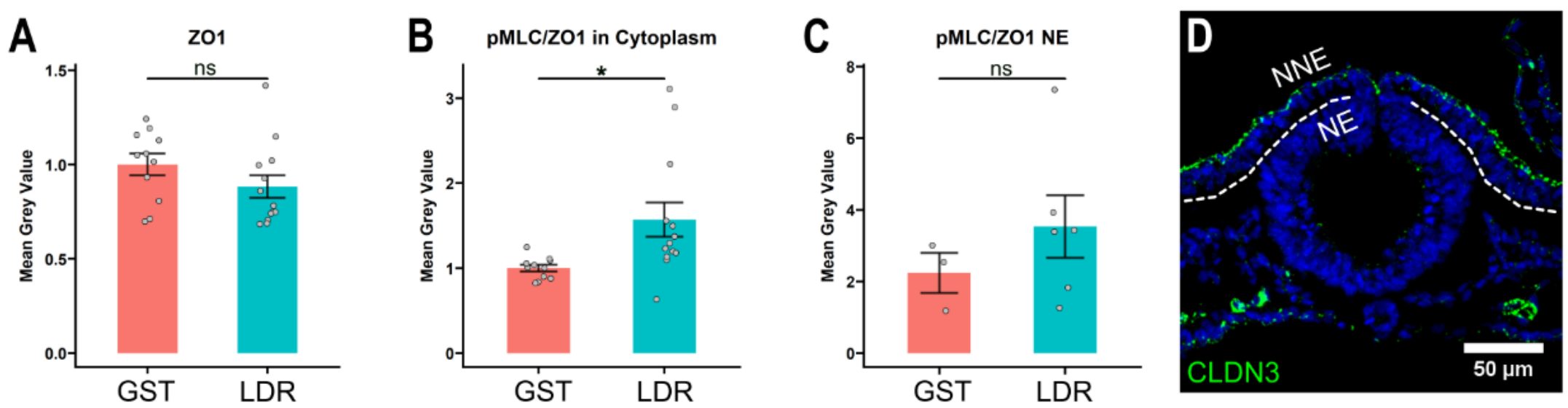

Fig. S3. Further analysis of pMLC expression in CLDN3-depleted chick embryos

A)Quantification of ZO1 fluorescence in control (GST; n=12) and CLDN3-depleted (LDR; n=11) embryos. B) Ratio of pMLC/ZO1 in the cytoplasmic region of cells in control (GST; n=12) and CLDN3-depleted (LDR; n=11) embryos. C) Ratio of pMLC/ZO1 in the neural ectoderm of control (GST; n=3) and CLDN3-depleted (LDR; n=6) embryos. In A, B, and C bars show the mean, error bars plot the standard error, and grey dots show the average measurement for each embryo. Statistical significance was evaluated with Student's T-test. * $p < 0.05$. D) CLDN3 immunofluorescence (green) in a transverse section through the spinal region of a control chick embryo (n=4). The white dashed line indicates the separation between non-neural ectoderm (NNE) and the neural ectoderm (NE). Statistical significance was evaluated with Student's T-test. * $p < 0.5$. Scale bar: D) 50 µm.

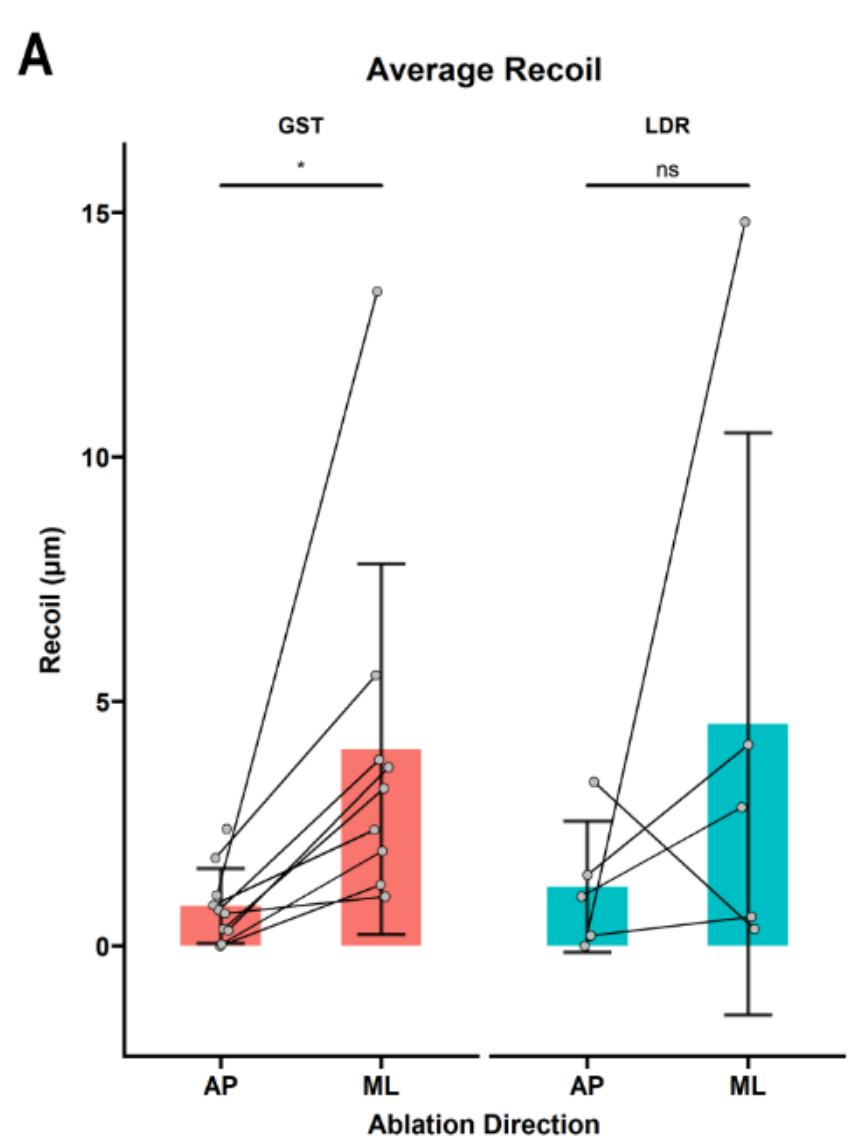

Fig. S4. Tension in the neural ectoderm is anisotropic.

A) Quantification of recoil in the neural ectoderm of control (GST; n=9) and CLDN3-depleted (LDR; n=5) embryos. Ablations in the medial-lateral (ML) and anterior-posterior (AP) direction were compared. Bars represent the mean and error bars represent the standard deviation. Points represent the average per embryo, and lines connect measurements made on the same embryo. Statistical significance was assessed with paired Student's T-test between ML and AP directed ablations. * $p < 0.05$.

Journal of Cell Science • Supplementary information

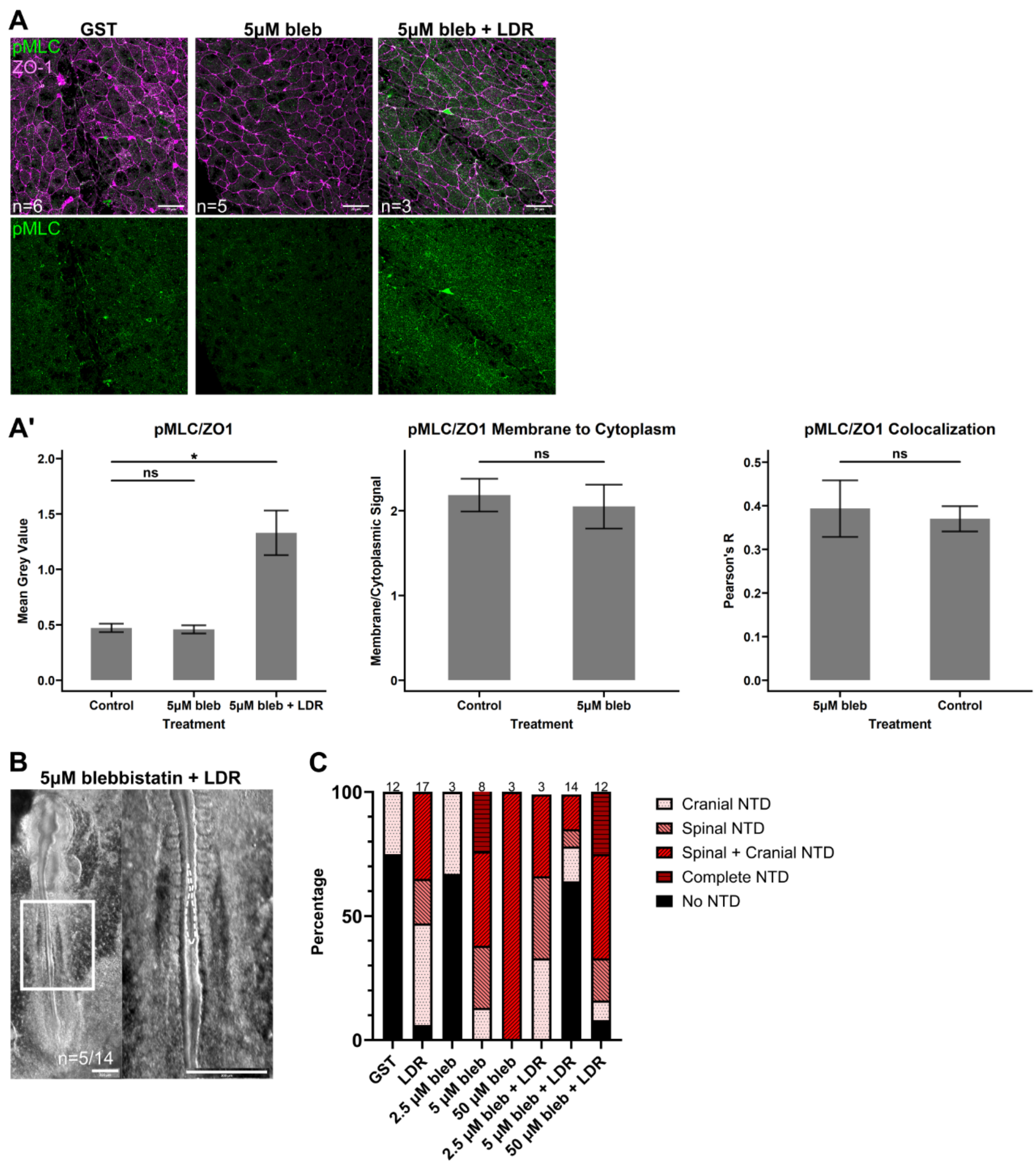

Fig. S5. Analysis of pMLC expression and classification of NTDs in blebbistatin-treated embryos.

A) Representative immunofluorescence surface-projected images of ZO1 (magenta) and pMLC (green) in embryos treated with GST + DMSO (GST; n=6), 5 μM blebbistatin (bleb; n=5), or 5μM blebbistatin + 400μg/mL LDR (bleb + LDR; n=3) A') Quantification of pMLC/ZO1 signal intensity in the non-neural ectoderm, the ratio of pMLC/ZO1 signal in the membrane/cytoplasm, and Pearson's colocalization between pMLC and ZO1. Bars show the mean and error bars show standard error. B) Representative images of an embryo treated with 5μM blebbistatin + 400μg/mL LDR (n=14) with a NTD (n=5/14). C) Quantification of NTD location (percentage) with total number of embryos for each treatment shown at the top of the bars. Statistical significance in A' was evaluated with Student's T-test. ns: not significant, * p< 0.5. Scale bars: A) 20μm, B) 200 μm.

Journal of Cell Science • Supplementary information

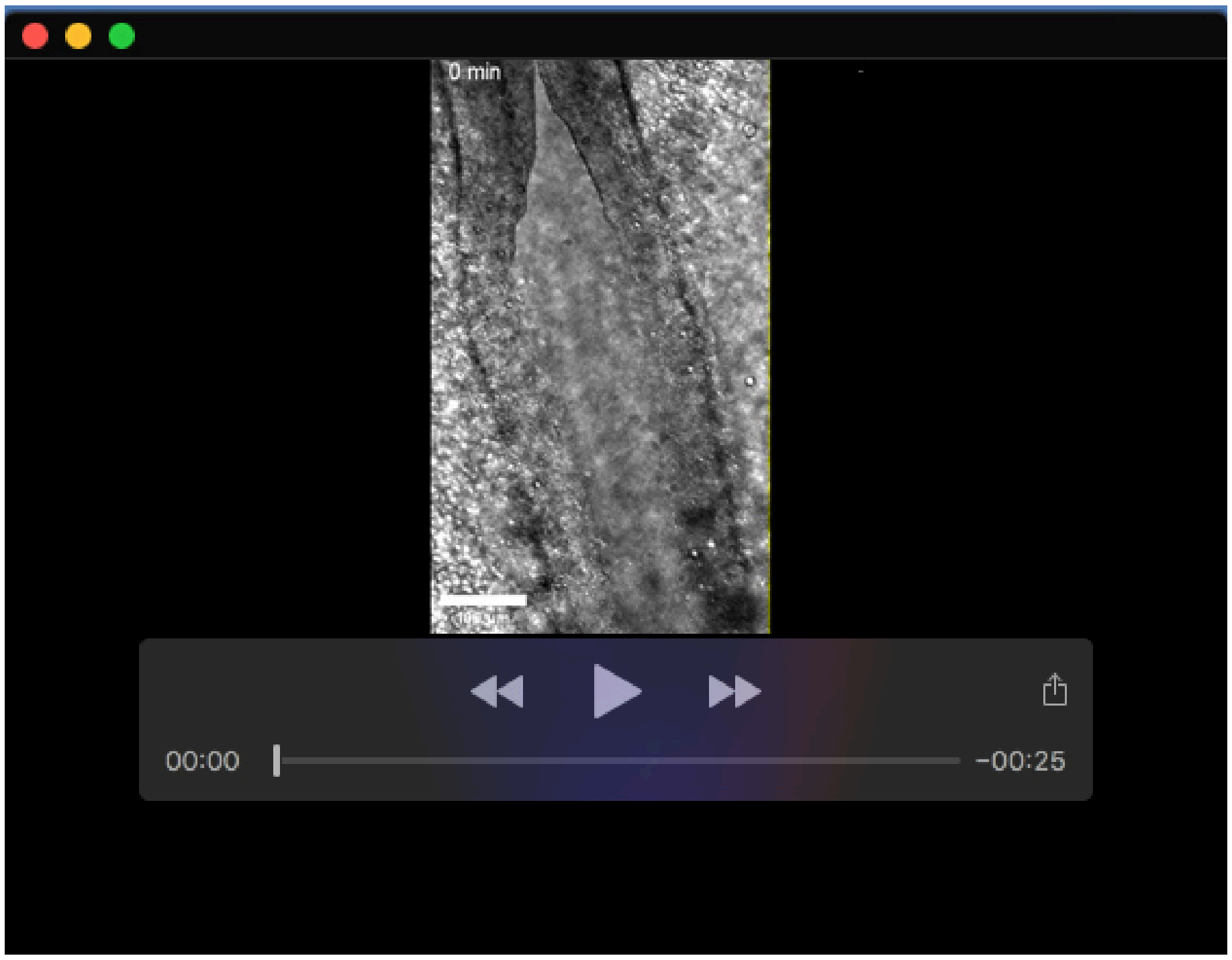

Movie 1. Live imaging of the formation of the RCNP in a control chick embryo.
Representative movie of RCNP formation in control embryos (n=5). The embryo is positioned so anterior is towards the "top". Time in minutes shown in upper left corner. Scale bar: 100μm

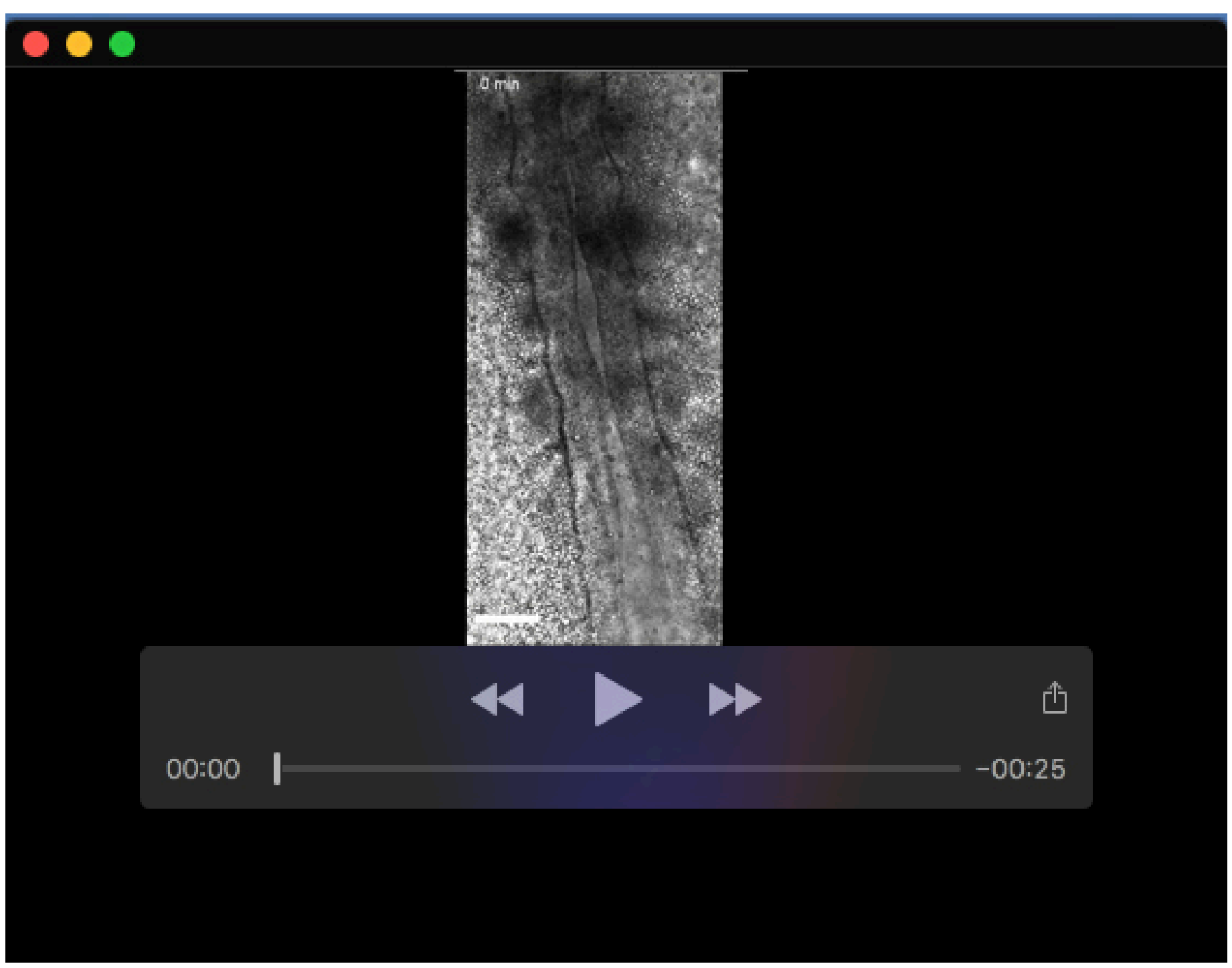

Movie 2. Live imaging of buttoning in the spinal region of a control chick embryo.

Supplemental movie 1 embryo was re-positioned to view the spinal region. The embryo is positioned so anterior is towards the "top". Time in minutes shown in upper left corner. Scale bar: 100μm.

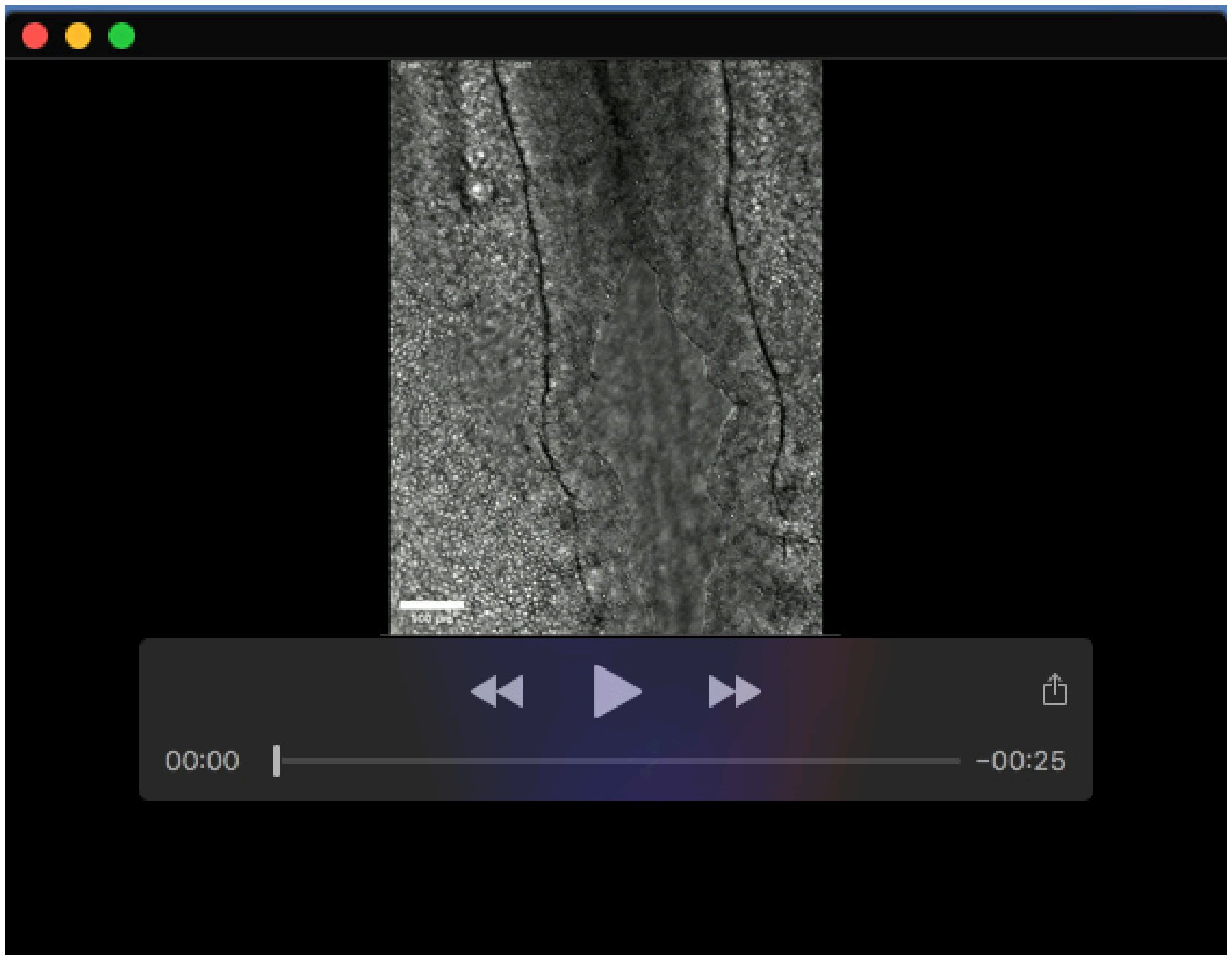

Movie 3. Live imaging of the RCNP in a GST-treated embryo.

Representative movie of control GST-treated embryos (n=5) used for analysis of closure rates in the RCNP. The embryo is positioned so anterior is towards the “top”. Time in minutes shown in upper left corner. Scale bar: 100µm.

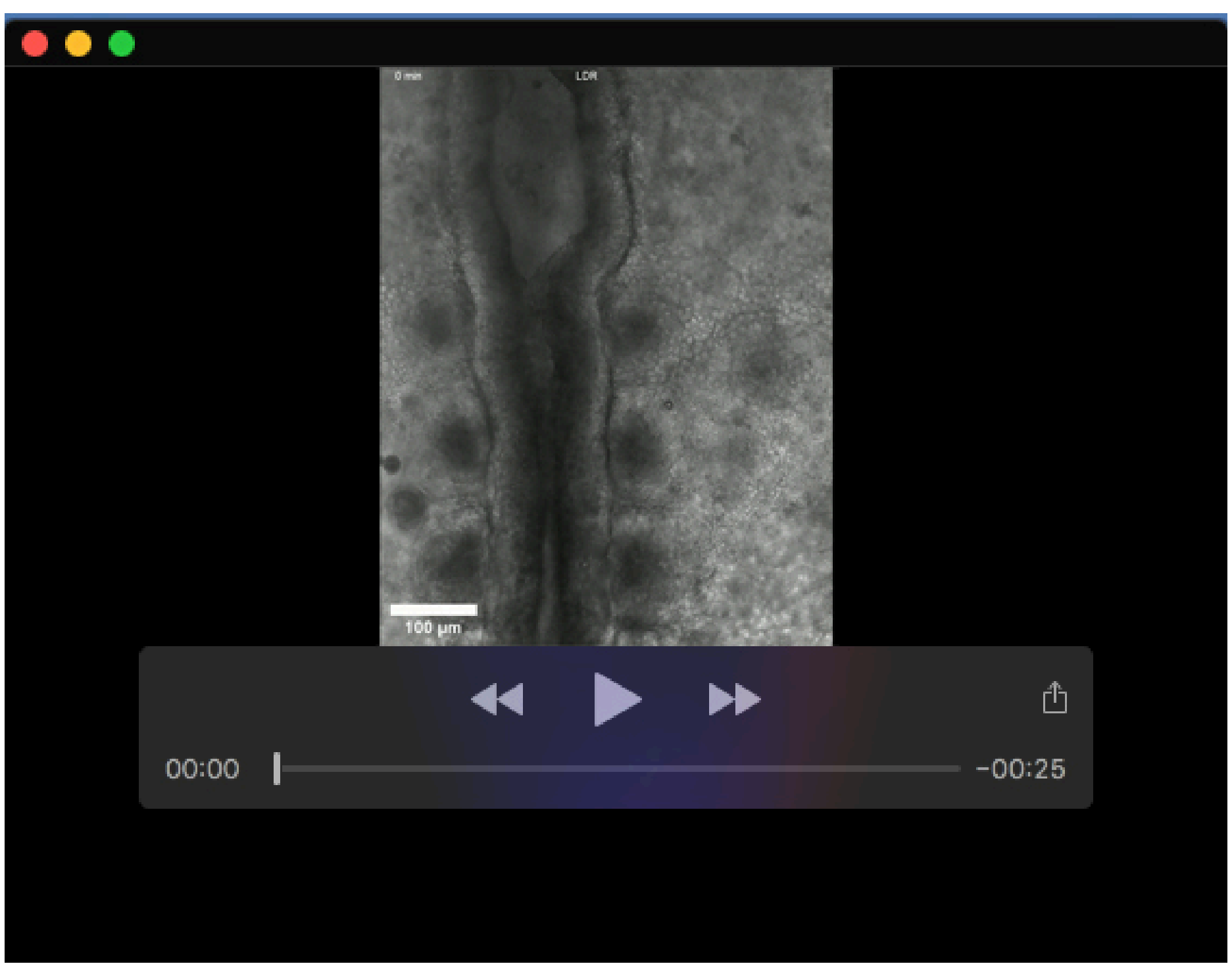

Movie 4. Live imaging of the RCNP in a LDR-treated embryo.

Representative movie of CLDN3-depleted LDR-treated embryos (n=4) used for analysis of closure rates in the RCNP. The embryo is positioned so anterior is towards the “top”. Time in minutes shown in upper left corner. Scale bar: 100µm.
