# Supplementary material for: Increased tissue tension caused by depletion of CLDN3 in the non-neural ectoderm causes neural tube defects in chick embryos
Source: J Cell Sci. 2026 Sep 25;139(18):jcs264758. doi: 10.1242/jcs.264758 (PMC13680966; doi:10.1242/jcs.264758)
Supplement: Supplementary file 2 [file joces-139-264758_review_history.pdf]

Journal of Cell Science

# Increased tissue tension caused by depletion of CLDN3 in the non-neural ectoderm causes neural tube defects in chick embryos

Elizabeth-Ann Legere, Marie Dumont, Jordan A. Dias, Yojiro Yamanaka, Gabriel L. Galea and Aimee K. Ryan
DOI: 10.1242/jcs.264758

**Editor**: Kathleen Green

**Review timeline**

| | |
|---|---|
| Original submission: | 29 January 2026 |
| Editorial decision: | 12 March 2026 |
| First revision received: | 2 July 2026 |
| Accepted: | 12 August 2026 |

---

**Original submission**

First decision letter

MS ID#: jcs.264758

MS TITLE: Increased tissue tension caused by depletion of CLDN3 in the non-neural ectoderm causes neural tube defects in chick embryos

AUTHORS: Elizabeth-Ann Legere; Marie Dumont; Yojiro L. Yamanaka; Gabriel L. Galea; Aimee K Ryan

ARTICLE TYPE: Research Article

Dear Dr Ryan,

We have now reached a decision on the above manuscript.

As you will see, the reviewers raise a number of substantial criticisms that prevent me from accepting the paper at this stage. They suggest, however, that a revised version might prove acceptable, if you can address their concerns. If you think that you can deal satisfactorily with the criticisms on revision, I would be pleased to see a revised manuscript. We would then return it to the reviewers.

Reviewer 1

The manuscript investigates the mechanism of CLDN3 function during neural tube closure in chick. The authors' main findings are: 1) the buttoning mode of closure is mechanistically distinct from zipping, 2) CLDN3 depletion specifically inhibits the buttoning mode of closure, 3) CLDN3 suppressed pMLC activation and tension in the non-neural ectoderm.

Overall, the manuscript provides new insights into the mechanism of neural tube closure. However, some of the conclusions are not sufficiently supported by the data and/or need better statistical comparisons.

Major comments:

© 2026. Published by The Company of Biologists under the terms of the Creative Commons Attribution License (https://creativecommons.org/licenses/by/4.0/).

1) Fig. 2: These cutting experiments need to have statistical comparisons. There are low numbers of embryos for some categories (i.e., 'Cut spinal'). In addition, some of the control situations have high numbers of NTDs (p. 7, line 31, n=2/4). It should be made more clear what the direct comparisons are for controls at each axial position.
2) Fig. 4B: The height of neural ectoderm cells in the tube looks different and the image doesn't clearly show where non-neural ectoderm is. Not all the images are surface projected, this should be noted in the figure legend. Authors need to describe the depth of the surface projection and how they distinguish non-neural and neural ectoderm for pMLC analysis.
3) Supplemental Fig. 5: Given that blebbistatin blocks myosin II heads in a low actin affinity state, one would expect that it would affect the localization of pMLC (PMID: 15205456). The effects of blebbistatin on non-neural ectoderm and neural ectoderm pMLC localization should be described to understand this phenotype. It is unclear why globally inhibiting myosin II would rescue the CLDN3-depletion phenotype, but analyzing the effects of blebbistatin on the different tissues, could help elucidate this.

Minor comments:
1) p. 3, Line 2-6: Cell division and cell division orientation should be mentioned as cell behaviors that contribute to neural tube development.

2) p. 5, Line 17-19: membrane blebs are also strongly correlated with contractility (PMID: 21822289).

3) Figure 1D2: Can't see the rosette because the signal is oversaturated.

4) Figure 3: Consistent terminology should be used between CLDN3-depleted vs. LDR throughout the manuscript.

5) p. 9, Line 20-21: There is a typo; one non-neural ectoderm should be neural ectoderm.

6) p. 10, Line 14: Should also include this reference for laser cutting - PMID: 12574496.

7) Fig. 5A: Should show the direction of the tissue recoil measured in Fig. 5A'.

8) Fig. 7: Inactive myosin II is in a folded conformation, and activated myosin II forms bipolar filaments (PMID: 18427938). Should redraw them more appropriately.

Reviewer 2

This manuscript builds on the authors' previous study showing that depletion of the tight junction protein CLDN3 in the non-neural ectoderm causes neural tube defects and alters epithelial polarity and cytoskeletal organization, including changes in PAR3 localization, reduced bicellular F-actin, and smaller apical surfaces. The present work extends these findings by examining the mechanical consequences of CLDN3 depletion during neural tube closure. The authors show that CLDN3 depletion slows spinal neural fold fusion, increases pMLC levels in the non-neural ectoderm, elevates tissue tension measured by laser ablation, and that partial inhibition of myosin II can rescue the phenotype. Together, these results support a model in which CLDN3 helps regulate actomyosin-dependent mechanical tension in the non-neural ectoderm during neural fold fusion.

Overall, the mechanical analysis is convincing, and the results appear compelling. The work provides an interesting perspective on the role of tissue tension during neural fold fusion. However, the manuscript would benefit from substantial improvement in presentation and clarity. Tightening the narrative, strengthening a few points mentioned below, and improving figure presentation would significantly strengthen the manuscript.

Major Comments:

1. Clarification of the "buttoning vs. zippering" section

The first results section attended to demonstrate that neural fold fusion in chick occurs through distinct mechanisms in cranial and spinal regions. However, this distinction is already described in

© 2026. Published by The Company of Biologists under the terms of the Creative Commons Attribution License (https://creativecommons.org/licenses/by/4.0/).

the introduction as established knowledge from previous work. It is therefore unclear what new insight this section provides. While this characterization provides useful context for the study, the narrative could be streamlined and more clearly connected to the central question of the manuscript.

2. Mechanistic link between polarity defects and increased contractility

The manuscript would benefit from a stronger mechanistic link between the elevated contractility reported here and the polarity changes described in the authors' previous work. In that study, CLDN3 depletion altered the localization of polarity proteins such as PAR3 and reduced bicellular F-actin. In the current manuscript, CLDN3 depletion leads to increased apical pMLC and tissue tension, but the relationship between these observations is not explored.

It would be interesting to examine whether CLDN3 depletion also alters apical actin organization or the distribution of cadherin. Such analyses could help bridge the polarity defects observed previously with the increased contractility described in the current study and strengthen the proposed mechanical model with a clearer molecular mechanism.

3. Use of ZO1 as a normalization reference

The authors use ZO1 as an unchanged reference marker. However, since CLDN3 depletion directly perturbs tight junction composition, I am not fully convinced that ZO1 provides a sufficiently independent normalization control in this context. Although the authors state that ZO1 remains unchanged, in this manuscript (Supp. Fig. 2A) and in their previous publication (e.g., Fig. 5A), the ZO1 signal appears noticeably increased under CLDN3 depletion, which makes me wonder how applicable this marker is for this analysis. It may be worth considering whether a different membrane marker could provide a more independent reference.

4. Local mechanical perturbation experiment

The rescue of the phenotype by low-dose blebbistatin provides important support for the role of actomyosin-dependent tension. However, since this is a global pharmacological treatment, the conclusions could be further strengthened by a more localized mechanical perturbation. For example, the authors could consider performing a similar cutting experiment to that shown in Figure 2, but placed laterally in the non-neural ectoderm or near the neural fold edge. If local release of tension in this region improves neural tube closure in CLDN3-depleted embryos, this would provide additional support for the proposed mechanical model.

Minor Comments:

5. Spatial specificity of the phenotype

The authors report that CLDN3 depletion primarily affects the spinal "buttoning" phase of neural fold fusion, while cranial zippering appears largely unaffected. I therefore wonder whether the authors have examined CLDN3 expression along the cranial-caudal axis, and whether regional differences in expression or localization might contribute to this spatial specificity.

6. Figures and schematic illustrations

I found several aspects of the figures and schematic illustrations difficult to interpret and have a number of questions and requests for clarification.

a. Figure 1A (insets 1-3): The schematic does not appear to reflect the realistic cell size and shape observed in the tissue and would benefit from being redrawn.

b. Figure 1C: The time labeling is somewhat confusing. A more standard time-stamp format may help. In addition, the text states a 20-minute interval between T5 and T6, which does not appear consistent with the 5-minute intervals shown in panel C".

© 2026. Published by The Company of Biologists under the terms of the Creative Commons Attribution License (https://creativecommons.org/licenses/by/4.0/).

c. Figure 2B & C (left panels): These are supposed to represent different stages, but the illustrations appear identical. In addition, in Fig. 2B the cut does not appear to be placed at the fifth somite as described.

d. Figure 3C: The authors state that "There was no change in the ratio of anterior-posterior to medial-lateral RCNP lengths between control and CLDN3-depleted embryos" and that "There were no observed changes in the initial formation of button contact points." However, the RCNP region in CLDN3-depleted embryos appears substantially smaller than the control at 30 minutes, and the neural tube also appears narrower. Some clarification would be helpful.

e. Figure 7: The model includes apical actin, but no corresponding data are shown in the manuscript.

7. Sentences that may require correction

a. "moment of the elevated neural folds at the dorsal midline to meet in the dorsal mid-line, stabilization at points of contact, and closure".

b. "CLDN3 is expressed throughout all cells of the non-neural ectoderm but not in the non-neural ectoderm."

8. Statements that may require adjustment in tone

a. "We observed that beginning at HH8 the elevated unfused neural folds appeared 'wavy' and periodically bulged out (Fig. 1C, D)." The periodicity is not clearly demonstrated in Fig. 1C, D.

b. " Our data also suggested that bulging of the neural folds is not driven by specific actomyosin constriction at the neural fold edge, as there was no enrichment of F-actin at unfused neural fold edges (n=7) (Figure 1D)." However, I see the opposite in the region more posterior to the outlined region.

---

**First revision**

<u>Author response to reviewers' comments</u>

We would like to thank the reviewers for their constructive feedback on our manuscript.

The manuscript has been revised to improve clarity and add additional information as requested by the reviewers. Also, we have included additional data to further examine the effect of surgically cutting the neural folds, and to increase the sample size for the blebbistatin-treatments and new data on the effect of blebbistatin on apical cell area in blebbistatin-treated control and CLDN3-depleted embryos. Below we provide our point-by-point response to the reviewers' comments.

**Reviewer 1:**

**Major comments:**
**1) Fig. 2:** These cutting experiments need to have statistical comparisons. There are low numbers of embryos for some categories (i.e., 'Cut spinal'). In addition, some of the control situations have high numbers of NTDs (p. 7, line 31, n=2/4). It should be made more clear what the direct comparisons are for controls at each axial position.

***Response:***
*We performed additional experiments confirming that cuts within the posterior neuropore stop zippering in all embryos, in contrast with more rostral cuts in the region of buttoning where closure continues despite these cuts. For this series of experiments, embryos were collected on filter paper supports and then an additional filter paper support was added so that the embryo*

© 2026. Published by The Company of Biologists under the terms of the Creative Commons Attribution License (https://creativecommons.org/licenses/by/4.0/). 

*was sandwiched between 2 filter paper supports to allow us to culture them* ex ovo *dorsal side up on agar albumen plates. In the original series of experiments, we observed that ~50% of our control embryos from which the vitelline membrane was removed had open posterior neuropores.*

*To investigate this further and increase the sample size, we repeated the experiments. Again, we found that removing the vitelline membrane causes NTDs in the posterior neuropore in 52% of embryos (n=15/31). However, we found a significant further increase in NTDs in the posterior neuropore in embryos cut in the posterior neuropore region (n=6/6, p=0.02) compared to removal of the posterior neuropore vitelline membrane alone. In this series of experiments, we included an unmanipulated control group, where the embryos were cultured as described but the vitelline membrane was not removed. We found that simply culturing the embryos ventral side down (the method used for all cutting experiments) also results in NTDs in the posterior neuropore in 50% of embryos (n=13/24) which is not significantly different from uncut embryos where the vitelline membrane is removed. These data are included in lines 20-24 on page 8 of the revised manuscript. Indeed, others have also suggested that this culturing technique increases vitelline membrane tension and causes NTDs in the posterior region (Kunz et al., 2023). This reinforces the idea that spinal neural tube fusion and closure of the posterior neuropore are regulated differently.*

*These data have been updated in the figure. We agree that by combining the controls for the cut at mid-brain/spinal regions and the controls for cut at the posterior neuropore (vitelline removed over posterior neuropore) in the same graph was inaccurate and we have separated the data into two graphs (now Figure 2B and D).*

**2) Fig. 4B:** The height of neural ectoderm cells in the tube looks different and the image doesn't clearly show where non-neural ectoderm is. Not all the images are surface projected, this should be noted in the figure legend. Authors need to describe the depth of the surface projection and how they distinguish non-neural and neural ectoderm for pMLC analysis.

***Response:***
*We did not observe any differences in pMLC signal in the neural ectoderm between control and CLDN3-depleted embryos (Supp. Figure 3C) and focused our imaging and analysis on the non-neural ectoderm (tissue labels added to Figure 4). This is supported by our finding of equivalent recoil following laser ablation in the neural ectoderm of control and CLDN3-depleted embryos (Supp. Figure 4). In our previous publication (Legere et al., 2024) we sectioned control and CLDN3-depleted embryos and did not observe any overt differences in tissue thickness of non-neural ectoderm or neural ectoderm.*

*We added details about projection method used where they were missing in figure legends (Fig. 5, 6, S5).*

*p. 25, Line 1-4: More information was added to methods about settings and strategies used in SurfacePeeler macro with settings: gaussian blur = 2, default threshold, -2 to 2 pixel thickness (~2μm). Thickness was set to capture apical surface of non-neural ectoderm using ZO1 signal on orthogonal views. Analysis was primarily conducted on non-neural ectoderm, distinguished from the neural ectoderm by larger apical cell areas.*

**3) Supplemental Fig. 5:** Given that blebbistatin blocks myosin II heads in a low actin affinity state, one would expect that it would affect the localization of pMLC (PMID: 15205456). The effects of blebbistatin on non-neural ectoderm and neural ectoderm pMLC localization should be described to understand this phenotype. It is unclear why globally inhibiting myosin II would rescue the CLDN3-depletion phenotype, but analyzing the effects of blebbistatin on the different tissues, could help elucidate this.

***Response:***
*The effect of blebbistatin on the localization of pMLC seems to be context dependent as we and others (Berger et al., 2018; Escuin et al., 2015; Hirano and Hirano, 2016) have observed no effect. We observed no change in pMLC localization after blebbistatin treatment, measured by the ratio of pMLC/ZO1 mean intensity at the membrane to the cytoplasm (DMSO: 2.18 ± 0.33, blebbistatin: 2.05 ± 0.44, p-value=0.69), or in the Pearson's colocalization of pMLC to ZO1 signal (DMSO: 0.37 ±*

© 2026. Published by The Company of Biologists under the terms of the Creative Commons Attribution License (https://creativecommons.org/licenses/by/4.0/). 

*0.05, blebbistatin: 0.39 ± 0.11, p-value = 0.76). We have updated Supplemental Figure 5 with this data.*

*We observed no change in pMLC/ZO1 mean intensity in the neural ectoderm of blebbistatin-treated embryos (DMSO: 0.51 ± 0.12, blebbistatin: 0.62 ± 0.19, p-value = 0.45).*

*We performed this experiment based on previous observations that blebbistatin reduces tension in the surface ectoderm in the mouse (Nikolopoulou et al., 2019). Our goal was to use it to counteract the increase in tension observed in CLDN3-depleted embryos. Indeed we found that a moderate 5μM dose of blebbistatin that caused NTDs on its own, led to a decreased incidence of NTDs in CLDN3-depleted (LDR-treated embryos).*

*As the laser ablation experiments were only possibly through a travel grant supported by an on-site collaboration, we were unable to measure non-neural ectodermal tension in the blebbistatin-treated embryos. However, to confirm that the rescue of CLDN3-depleted NTDs by blebbistatin was due to decreasing non-neural ectodermal tension/actomyosin contraction we used a previously reported observation that the average non-neural ectodermal apical cell area was smaller in CLDN3-depleted cells as a read-out of altered apical actomyosin dynamics (Legere et al., 2024). Apical cell areas were segmented from surface-projected ZO1 immunofluorescence images, and we repeated the finding that CLDN3-depleted embryos (n=12) had smaller average apical cell areas compared to control embryos (n=9) (GST + DMSO: 87 $\mu m^2$ ± 18, LDR + DMSO: 70 $\mu m^2$ ± 10, p = 0.01). Treating CLDN3-depleted embryos with 5μM blebbistatin (n=10) increased average apical cell areas to baseline (LDR + DMSO: 70 $\mu m^2$ ± 10, LDR + 5μM blebbistatin: 84 $\mu m^2$ ± 18, p = 0.032). These data have been added to Fig. 6E, F and the figure legend and results section have been updated.*

**Minor comments:**
1) p. 3, Line 2-6: Cell division and cell division orientation should be mentioned as cell behaviors that contribute to neural tube development.

***Response:*** *p. 23 Line 13: Added "oriented cell divisions" to description of epithelial cell behaviors in neural tube development and updated references with "(Kieserman and Wallingford, 2009)".*

2) p. 5, Line 17-19: membrane blebs are also strongly correlated with contractility (PMID: 21822289).

***Response:*** *p. 6, Lines 1-4: Added "and/or increased contractility" with reference "(Sedzinski, 2011)" to description of membrane blebs.*

3) Figure 1D2: Can't see the rosette because the signal is oversaturated.

***Response:*** *To better show rosette shape we traced surrounding cells with dashed lines in Figure 1D2.*

4) Figure 3: Consistent terminology should be used between CLDN3-depleted vs. LDR throughout the manuscript.

***Response:*** *We understand the confusion in using two different terminologies. Our intent was to use CLDN3-depleted as a descriptive terminology within the text and LDR as the treatment terminology within the figures. We have edited the text to better reflect these distinctions and added a clarifying sentence p.9, Lines 4-6. "**We refer to LDR-treated embryos as CLDN3-depleted and the GST-treated embryos as control embryos throughout the text, and use LDR and GST labels, respectively in figures**."*

5) p. 9, Line 20-21: There is a typo; one non-neural ectoderm should be neural ectoderm.

***Response:*** *fixed error p.10, line 19*

6) p. 10, Line 14: Should also include this reference for laser cutting - PMID: 12574496.

***Response:*** *p. 11, Line 7: Added reference "(Hutson, 2003)" to laser cutting description.*

© 2026. Published by The Company of Biologists under the terms of the Creative Commons Attribution License (https://creativecommons.org/licenses/by/4.0/).

7) Fig. 5A: Should show the direction of the tissue recoil measured in Fig. 5A'.
***Response:*** *Updated Figure 5A' to show direction of tension measurements.*

8) Fig. 7: Inactive myosin II is in a folded conformation, and activated myosin II forms bipolar filaments (PMID: 18427938). Should redraw them more appropriately.

***Response:*** *Updated Figure 7 with redrawn myosin II to show active unfolded bipolar filaments and phosphorylation.*

**Reviewer 2:**

This manuscript builds on the authors' previous study showing that depletion of the tight junction protein CLDN3 in the non-neural ectoderm causes neural tube defects and alters epithelial polarity and cytoskeletal organization, including changes in PAR3 localization, reduced bicellular F-actin, and smaller apical surfaces. The present work extends these findings by examining the mechanical consequences of CLDN3 depletion during neural tube closure. The authors show that CLDN3 depletion slows spinal neural fold fusion, increases pMLC levels in the non-neural ectoderm, elevates tissue tension measured by laser ablation, and that partial inhibition of myosin II can rescue the phenotype. Together, these results support a model in which CLDN3 helps regulate actomyosin-dependent mechanical tension in the non-neural ectoderm during neural fold fusion.

Overall, the mechanical analysis is convincing, and the results appear compelling. The work provides an interesting perspective on the role of tissue tension during neural fold fusion. However, the manuscript would benefit from substantial improvement in presentation and clarity. Tightening the narrative, strengthening a few points mentioned below, and improving figure presentation would significantly strengthen the manuscript.

**Major Comments:**

**1. Clarification of the "buttoning vs. zippering" section**

The first results section attended to demonstrate that neural fold fusion in chick occurs through distinct mechanisms in cranial and spinal regions. However, this distinction is already described in the introduction as established knowledge from previous work. It is therefore unclear what new insight this section provides. While this characterization provides useful context for the study, the narrative could be streamlined and more clearly connected to the central question of the manuscript.

***Response:*** *Closure of the spinal neural tube by buttoning was first proposed by van Straaten and colleagues (1993, 1996) based on analysis of static images. While we agree that the live imaging characterization of chick spinal neural fold fusion in the first section of the Results is lengthy, this is the first live imaging analysis showing the differences between chick neural fold fusion in the cranial and spinal regions. Therefore, we felt it was important to provide these details and the timing. We believe that these data will be important for the field.*

**2. Mechanistic link between polarity defects and increased contractility**

The manuscript would benefit from a stronger mechanistic link between the elevated contractility reported here and the polarity changes described in the authors' previous work. In that study, CLDN3 depletion altered the localization of polarity proteins such as PAR3 and reduced bicellular F-actin. In the current manuscript, CLDN3 depletion leads to increased apical pMLC and tissue tension, but the relationship between these observations is not explored.

It would be interesting to examine whether CLDN3 depletion also alters apical actin organization or the distribution of cadherin. Such analyses could help bridge the polarity defects observed previously with the increased contractility described in the current study and strengthen the proposed mechanical model with a clearer molecular mechanism.

© 2026. Published by The Company of Biologists under the terms of the Creative Commons Attribution License (https://creativecommons.org/licenses/by/4.0/). 

***Response:*** *We know from our previous work that E-cadherin's expression and localization pattern are not changed by depletion of CLDN3 (Legere et. al, 2024). We have expanded our discussion section with further ideas about the connection between CLDN3, apical polarity pathways, and cell contractility (p. 16, Lines 8-14). We agree that examining mechanistic links between cell contractility and apical polarity pathways is very interesting and we are pursuing this work by investigating the direct and indirect interactions between CLDN3 and other apical proteins to better understand of the molecular mechanisms underlying these cell behaviours.*

**3. Use of ZO1 as a normalization reference**

The authors use ZO1 as an unchanged reference marker. However, since CLDN3 depletion directly perturbs tight junction composition, I am not fully convinced that ZO1 provides a sufficiently independent normalization control in this context. Although the authors state that ZO1 remains unchanged, in this manuscript (Supp. Fig. 2A) and in their previous publication (e.g., Fig. 5A), the ZO1 signal appears noticeably increased under CLDN3 depletion, which makes me wonder how applicable this marker is for this analysis. It may be worth considering whether a different membrane marker could provide a more independent reference.

***Response:*** *Although we do observe expected signal variation, we have not observed consistent effects on ZO-1 signal after use of CLDN depleting peptides (Legere et. al, 2024), (Baumholtz et. al, 2017), and (Gamero-Estevez et. al, 2019). There is no statistical difference in ZO1 intensity when quantified in either this manuscript or the previous publication (Legere et. al, 2024) where we quantified two different ZO1 antibodies to confirm that there was no change in either. In our previous publication, we found that E-cadherin signal was also unchanged between control and CLDN3-depleted embryos, and when used as a reference marker to ZO1, there was no statistical difference. We decided to use ZO1 as a reference marker over E-cadherin in this current manuscript as it is a marker for apical tight junctions and would allow for observation of localization in orthogonal views. We also tested other antibodies that we could use as a reference tight junction membrane marker (ZO2/Occludin) but found them unreliable for use in chick embryos.*

*For the image (Supp. Figure 2A) we were focused on showing the quantification of cell counts and so the control and CLDN3-depleted images were from different immunofluorescence experiments. We understand the confusion that arose and have replaced the CLDN3-depleted image with one from the same experiment as the control.*

**4. Local mechanical perturbation experiment**

The rescue of the phenotype by low-dose blebbistatin provides important support for the role of actomyosin-dependent tension. However, since this is a global pharmacological treatment, the conclusions could be further strengthened by a more localized mechanical perturbation. For example, the authors could consider performing a similar cutting experiment to that shown in Figure 2, but placed laterally in the non-neural ectoderm or near the neural fold edge. If local release of tension in this region improves neural tube closure in CLDN3-depleted embryos, this would provide additional support for the proposed mechanical model.

***Response:*** *The experiment proposed is interesting, however with the quick initiation of wound healing in embryo tissue* (Lawson and England, 1998; Wyczalkowski et al., 2013), *the effect would not last the required ~4-8 hours to culture the embryo to the stage where we could access the presence of NTDs. Although global, we decided to use the targeted inhibition of myosin to show that decreasing the tension in CLDN3-depleted embryos can rescue the NTDs, and used the timing of dosage (HH7/8 – beginning of neural fold fusion) to restrict the effect to neural fold fusion and the role of the non-neural ectoderm. Additionally, the dorsal-up culture method used for cutting experiments is not amenable to treatment with the CLDN3-depleting peptide and we found that the embryos must be cultured dorsal-down to have the neural tube developing in contact with the LDR-containing agar-albumin plates.*

**Minor Comments:**

**5. Spatial specificity of the phenotype**

© 2026. Published by The Company of Biologists under the terms of the Creative Commons Attribution License (https://creativecommons.org/licenses/by/4.0/).

The authors report that CLDN3 depletion primarily affects the spinal "buttoning" phase of neural fold fusion, while cranial zippering appears largely unaffected. I therefore wonder whether the authors have examined CLDN3 expression along the cranial-caudal axis, and whether regional differences in expression or localization might contribute to this spatial specificity.

***Response:*** *That is a very interesting idea and something we have examined. Previously our lab and others have published an overview of claudin staining by in situ hybridization over chick development and showed that CLDN3 is expressed throughout the non-neural ectoderm with no overt differences in expression between cranial and spinal regions (Simard et al., 2006) (Collins et al., 2013) (Haworth et al., 2005). We have added further descriptions with references to introduction (p.5, Line 29-30). As a sanity check we compared ratios of CLDN3/ZO1 signal from immunofluorescence images of the non-neural ectoderm between cranial and spinal regions and found no statistical difference with paired Student's T-test (cranial: 734 ± 272, spinal: 608 ± 384, p-value = 0.37). Because these observations were previously published we have not included them in a supplemental figure.*

**6. Figures and schematic illustrations**

I found several aspects of the figures and schematic illustrations difficult to interpret and have a number of questions and requests for clarification.

**a. Figure 1A** (insets 1-3): The schematic does not appear to reflect the realistic cell size and shape observed in the tissue and would benefit from being redrawn.

***Response:*** *We kept the more cuboidal shape of the non-neural ectodermal cells but redrew the neural ectoderm to better reflect the elongated cell shapes.*

**b. Figure 1C:** The time labeling is somewhat confusing. A more standard time-stamp format may help. In addition, the text states a 20-minute interval between T5 and T6, which does not appear consistent with the 5-minute intervals shown in panel C".

***Response:*** *Added more standard time-stamp formats to images and corrected error with 5-minute intervals in Figure 1C".*

**c. Figure 2B & C (left panels):** These are supposed to represent different stages, but the illustrations appear identical. In addition, in Fig. 2B the cut does not appear to be placed at the fifth somite as described.

***Response:*** *Updated Figure 2B to more accurately place cut on schematic and replaced Figure 2C schematic to better represent the stage.*

**d. Figure 3C:** The authors state that "There was no change in the ratio of anterior-posterior to medial-lateral RCNP lengths between control and CLDN3-depleted embryos" and that "There were no observed changes in the initial formation of button contact points." However, the RCNP region in CLDN3-depleted embryos appears substantially smaller than the control at 30 minutes, and the neural tube also appears narrower. Some clarification would be helpful.

***Response:*** *The embryo used in Figure 3C for the GST control was one of the cases where the RCNP formed between the hindbrain and somite 3 compared to the hindbrain and somite 1, however this does not change our measurement of the rate of width between neural folds over time and did not affect the comparison of ratio of anterior-posterior to medial-lateral RCNP lengths between control and CLDN3-depleted embryos. To examine this further we measured the circularity (4π*area/perimeter^2, circularity closer to 1 = circle, closer to 0 = elongated) of the RCNPs on the fixed images and found no significant difference between control and CLDN3-depleted embryos (GST: 0.36 ± 0.2, LDR: 0.44 ± 0.1, p-value = 0.48). Data added to Supplemental Figure 2.*

**e. Figure 7:** The model includes apical actin, but no corresponding data are shown in the manuscript.

© 2026. Published by The Company of Biologists under the terms of the Creative Commons Attribution License (https://creativecommons.org/licenses/by/4.0/).

***Response:*** *In our previous publication we found that bicellular apical actin was decreased (Legere et al., 2024). We added references to this data in the Results (p. 10, Line 12) and Discussion (p. 17, Line 13).*

7. Sentences that may require correction
a. "moment of the elevated neural folds at the dorsal midline to meet in the dorsal mid-line, stabilization at points of contact, and closure".

***Response:*** *p. 3, Line 19-20: edited*

b. "CLDN3 is expressed throughout all cells of the non-neural ectoderm but not in the non-neural ectoderm."

***Response:*** *p. 10, Line 19: fixed error*

**8. Statements that may require adjustment in tone**

a. "We observed that beginning at HH8 the elevated unfused neural folds appeared 'wavy' and periodically bulged out (Fig. 1C, D)." The periodicity is not clearly demonstrated in Fig. 1C, D.

***Response:*** *p. 7, Line 18-19: We apologize for the confusion and have removed "periodically bulged out".*

b. " Our data also suggested that bulging of the neural folds is not driven by specific actomyosin constriction at the neural fold edge, as there was no enrichment of F-actin at unfused neural fold edges (n=7) (Figure 1D)." However, I see the opposite in the region more posterior to the outlined region.

***Response:*** *p. 7, Line 21-23: Added further details about where we expect to see enrichment of F-actin (when neural folds touch during active fusion as you can see in the region immediately posterior to the outline region in Figure 1D to better compare to where we do not (when neural folds are not in contact).*

---

Second decision letter

MS ID#: jcs.264758R1

MS Title: Increased tissue tension caused by depletion of CLDN3 in the non-neural ectoderm causes neural tube defects in chick embryos

Authors: Elizabeth-Ann Legere; Marie Dumont; Jordan A Dias; Yojiro L. Yamanaka; Gabriel L. Galea; Aimee K Ryan

Article Type: Research Article

Dear Dr Ryan,

I am happy to tell you that your manuscript has been accepted for publication in Journal of Cell Science, pending standard publication integrity checks.

Reviewer 1

SUMMARY OF THE ADVANCE MADE IN THIS PAPER AND ITS POTENTIAL SIGNIFICANCE TO THE FIELD

The manuscript investigates the mechanism of CLDN3 function during neural tube closure in chick. The authors' main findings are: 1) the buttoning mode of closure is mechanistically distinct from

© 2026. Published by The Company of Biologists under the terms of the Creative Commons Attribution License (https://creativecommons.org/licenses/by/4.0/).

zipping, 2) CLDN3 depletion specifically inhibits the buttoning mode of closure, 3) CLDN3 suppressed pMLC activation and tension in the non-neural ectoderm.

Overall, the manuscript provides new insights into the mechanism of neural tube closure.

SUGGESTIONS TO AUTHORS

The conclusions are now sufficiently supported by the data. The authors have addressed all my concerns.

Minor comments

Reviewer 2

We appreciate the authors' efforts to add detailed information while significantly improving the clarity of this work. All of my concerns from the previous review have been properly addressed.

© 2026. Published by The Company of Biologists under the terms of the Creative Commons Attribution License (https://creativecommons.org/licenses/by/4.0/).
